# A systematic study on sustainable low carbon cement – Superplasticizer interaction: Fresh, mechanical, microstructural and durability characteristics

**DOI:** 10.1016/j.heliyon.2023.e19176

**Published:** 2023-08-21

**Authors:** Ishan Bhandari, Rajesh Kumar, A. Sofi, Nikhil Sanjay Nighot

**Affiliations:** a CSIR-Central Building Research Institute, Roorkee, Uttarakhand, 247 667, India; b Academy of Scientific and Innovative Research (AcSIR), Ghaziabad, 201 002, India; c Structural & Geotechnical Engineering, School of Civil Engineering, Vellore Institute of Technology, Vellore 632 014, India

**Keywords:** Supplementary cementitious material, Low carbon cement, Fly ash, Limestone, Calcined clay, Superplasticizer, Life cycle assessment

## Abstract

As governments around the world take on ambitious construction projects, from housing to infrastructure to transportation, the demand for cement is set to rise. It is anticipated that global cement production is set to achieve a compound annual growth rate of ∼5.1% for the years 2022–2025. The negative impact of cement production on the environment, such as carbon emissions and energy consumption, is also well known. This instigates the need to look for alternative and sustainable supplementary cementitious materials (SCMs) such as Fly ash (FA), Limestone (LS), Metakaolin (MK), Ground granulated blast furnace slag (GGBFS) and Silica fume (SF) which when blended with Portland clinker result in lower carbon emissions and better end products. With expanding cement demand, the need for chemical admixtures has also increased. This comprehensive study focuses on the compatibility of commercially available superplasticizers with SCMs blended low carbon cement and their influence on fresh and hardened properties along with microstructural and durability aspects. The chemistry of superplasticizers and how it effects the hydration mechanism of blended cement are also highlighted in detail. Moreover, the effect of different types of superplasticizers, their dosage, water binder ratio, and details of experiments used by other authors are also discussed and listed. As cementitious matrix containing any kind of SCM such as FA showed better environmental performance on the basis of life cycle assessment which was due to carbon emission factor (ξ_i_). For cement, ξ_i_ was 311.27 kg CO_2_-eq/t, whereas for FA it was much lower (8.70 kg CO_2_-eq/t). Based on this comprehensive literature review, current challenges for the utilisation of waste SCMs incorporating superplasticizers along with research gap have been identified. Apart from this, the ongoing research work on the effect of chemical and mineral admixture on Limestone-calcined clay cement (LC^3^) using statistical modelling to optimize the mix is also discussed. It was observed that the use of a specific type of mineral admixture with a superplasticizer inversely affected the mechanical properties like compressive strength and modulus of rupture but improved the water-binder ratio, porosity, and water absorption.

## Highlights

• Synergic interactions between different SCMs and OPC during hydration are presented.
• Interaction of superplasticizer with the cementitious mix are presented.
• Compatibility of commercial superplasticizers with low carbon cement is discussed.
• Effective use of modified superplasticizer is under-represented in the field of low carbon cements.
• Ongoing research work on use of superplasticizer and cellulose polymer is presented.

## Graphical abstract

**Figure:**
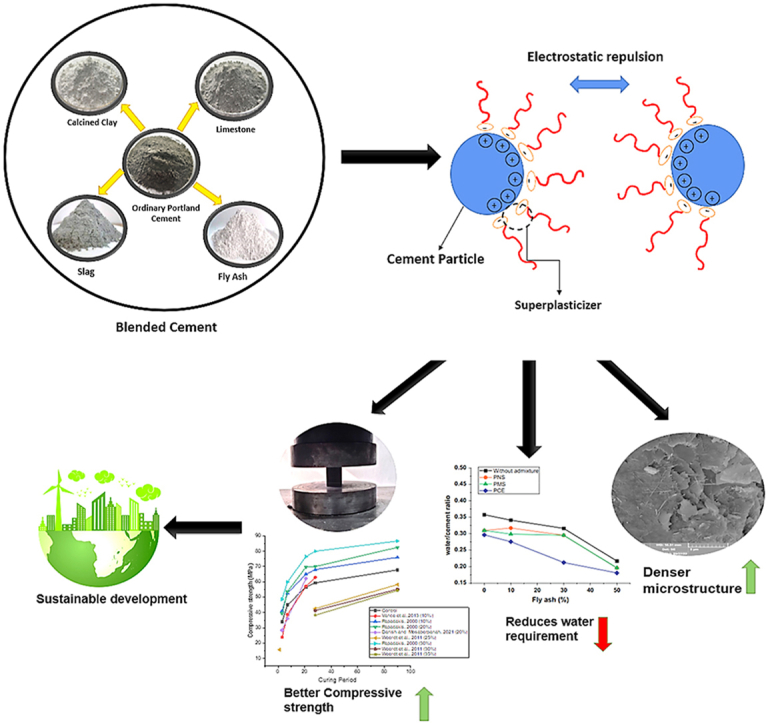

## List of abbreviations

SCMs: Supplementary cementitious materials
OPC: Ordinary Portland cement
FA: Fly ash
LS: Limestone
MK: Metakaolin
SF: Silica fume
GGBFS: Ground granulated blast furnace slag
LC^3^: Limestone-calcined clay cement
OPC: Ordinary Portland cement
C–S–H: Calcium silicate hydrate
PLC: Portland limestone cement
LCC: Low carbon cement
PCE: Polycarboxylate ether
PMS: Polymelamine sulfonate
PNS: Polynaphtalene Sulfonate
LS: Lignosulfonate
CH: Calcium hydroxide

## 1 Introduction

**Fig. 1:**
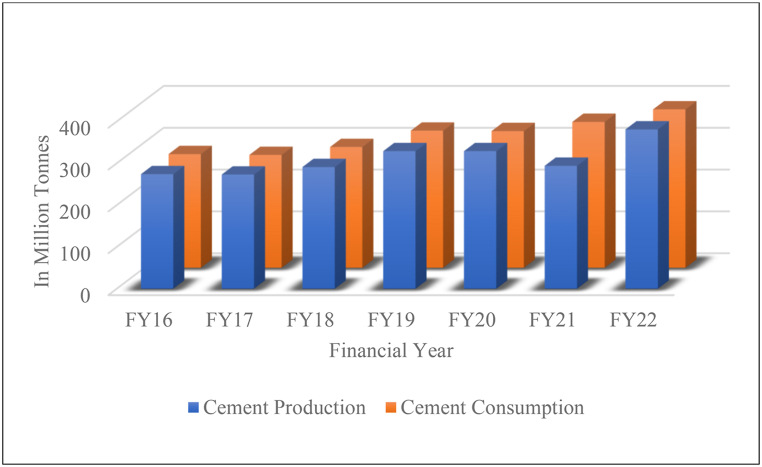
Year wise Cement Production and Consumption in India [5] (Accessed 20 Feb 2023).

Concrete has been used more than any other material in history, even more than water. Its high compressive strength, durability, resistance to environmental hazards, versatility and cost effectiveness have been the key driving factors of its success. An estimated 2 to 3% of the world's yearly energy use and 9 to 10% of industrial water are used by the concrete industry. The sector also generates 8–9% of the world's greenhouse gas emissions, or nearly 10 billion metric tonnes annually, more than any other sector and more than any other material [[1], [2], [3]]. Additionally, it is predicted that until 2050, the demand for concrete will increase, reaching 19 billion metric tonnes annually, due to an ever-increasing population and the need for housing and related infrastructure, particularly in developing nations. For over 200 years, Ordinary portland cement (OPC) has been the primary cementitious material for concrete production. The calcination of a few basic materials, including clay and limestone, at 1450 °C, is necessary for the production of OPC [4]. In order to produce 1 tonne of cement, this combustion process generates roughly 1 tonne of anthropogenic carbon dioxide. Compared to grinding, mixing, and transportation, clinkering requires more energy. The second-largest cement market, India, anticipates a 12% growth in cement production in the financial year 2023 as a result of higher government infrastructure spending and rural house building programmes [5]. Fig. 1 gives a glimpse of cement production and consumption over the past few years.

**Fig. 2:**
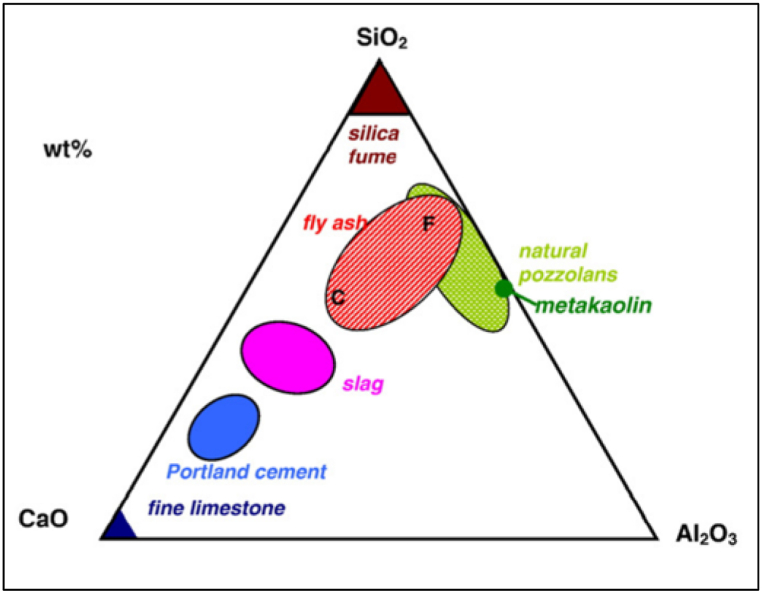
Ternary diagram of cementitious materials.

To mitigate the detrimental effect of cement production on the environment and energy consumption, two technologies are broadly adopted. While the first technology focuses on reducing energy requirements in the production of cement by substituting fossil fuel energy from renewable sources, the second suggests partial replacement of clinker with various supplementary cementitious materials (SCMs) such as fly ash (FA), limestone (LS), ground granulated blast furnace (GGBFS), metakaolin (MK), biomass ash, steel slag, etc. [6]. During the hydration of cement, silica and calcium compounds react with water to form C–S–H gel. By adding SCMs, the amorphous silica reacts with calcium hydroxide to form extra C–S–H gel [7]. The hydration of cement blended with SCMs is different from the hydration of OPC, as SCMs generally tend to have low calcium content (Fig. 2), which further effects their strength and durability properties. Clinker factor for cement is defined as percentage of clinker in cement. By using SCMs, the clinker factor can be reduced from 0.65 to 0.60 globally, which translates to a reduction of 2.9 × 10^9^ tonnes of CO_2_ global emissions [8,9]. SCMs also consume far less embodied energy, making them sustainable building materials. "Embodied energy (EE) is the energy consumed for raw material extraction, transportation, manufacture, assembly, installation, disassembly, and deconstruction for any product system over the duration of a product's life" [10]. Table 1 compares the embodied carbon dioxide (ECO_2_) for different SCMs.

**Table 1:** ECO_2_ of OPC, GGBS, PFA and limestone [6].

Materials	Embodied CO_2_ kg/tonne
OPC	910
FA	4
LS	71
GGBFS	72

To optimize the level of cement replacement, various types of binary and ternary blended cements are studied. With coal contributing around 40% to the world's electricity generation, fly ash continues to create a disposal problem due to its hazardous environmental implications. Nearly 800 Mt of FA is produced worldwide annually. Use of fly ash as replacement for cement from 15% to 30% has been extensively studied in recent decade [11]. According to ASTM C618–19, fly ash is classified as class C-fly ash (high calcium) and class F-fly ash (low calcium). Due to the high abundance of class F-fly ash, it has been focused on in this comprehensive review.

Concrete with fly ash has reduced early age strength development compared to plain concrete. This drawback of fly ash blended cement prevents its use in widespread application for example where early removal of formwork is needed or in prestress applications [[12], [13], [14]]. In case of GGBFS blended cement system, optimum level of replacement is suggested to by around 30–40%. At these replacement levels, GGBFS blended concrete gains strength faster than plain concrete; it has slightly lower compressive strength, but there is a significant reduction in total carbon dioxide emissions by nearly 30% [15]. Unfortunately, the supply of traditional SCMs such as fly ash or GGBFS is limited as it depends on the by-products of other industries, and thus it is not able to keep up with the rapidly increasing cement demands, especially in developing countries.

Limestone (LS) has been used as a filler in concrete, which has helped lower the cost of production and reduce carbon emissions from cement production. LS binds the extra water in concrete, which improves packing density. Although Portland limestone cement (PLC) has been studied extensively, but the pozzolanic activity of limestone itself is very low. Also, PLC showed a high amount of portlandite [16]. Whereas, when metakaolin is blended with PLC; the portlandite has been observed to decrease significantly from as early as 1 day.

Metakaolin clay is being produced when kaolinite clay is thermally treated at temperatures of 650–850 °C. The initial water removal from the interlayer is followed by dihydroxylation, which makes clay amorphous [17,18]. Clay particles have a layered structure made out of sheets of silicon and aluminium. Based on the stacking arrangement of sheets and the way in which layers are attached together, it dictates the formation of different clay minerals.

When Blast furnace slag, in a molten state, is rapidly cooled by immersion in cold water, a granular glassy material so formed is called Ground Granulated blast furnace slag (GGBFS). Similar to FA, GGBFS has also been used in concrete for years as a mineral admixture or as a partial replacement of portland cement (PC), which improves workability, increases slump, and provides better long-term properties, especially in self-compacting concrete (SCC).

As the availability of FA and GGBFS is limited, now-a-days calcined clay (Metakaolin) is attracting growing interest as an alternate SCM. The temperature required for calcination of clay is around 600–900 °C [19], which is less compared to portland cement production at around 1450 °C [4]. The global annual production of FA is 1 billion metric tonnes, LS is 338.55 million metric tonnes, MK is 6 billion metric tonnes and GGBFS is 360 billion metric tonnes [20,21]. As per ASTM C1240 - 20, ASTM C595 - 10, ASTM C618 - 19 and ASTM C989 – 19 [[22], [23], [24], [25]], the replacement levels of FA, LS, MK, and GGBFS range from 10% to 35%, 5%–25%, 5%–10%, and 40%–70%, respectively. As it can also be seen in Table 2, these are the replacement levels that are mostly used in research as well. From the review of various papers related to the topic, it has been observed that replacement levels for SCMs range from 5% to 50%. The most commonly used superplasticizer is PCE-based, and tests such as compressive strength, isothermal calorimetry, slump flow, X-ray diffraction, etc. are being conducted. Table 3 shows the chemical and physical composition of the SCMs mainly targeted for this study. It can be ascertained that FA mainly contained silicon dioxide, varying in proportion from 39.50% to 54.40%. LS contains calcium oxide as a primary oxide, ranging from 42.30% to 56.53%. MK primarily contains silicon dioxide and aluminium oxide, ranging from 49.50% to 51.40% and 40.25%–51.70%, respectively. The main oxide in GGBFS is silicon dioxide, ranging from 32.71% to 40.98%. Concrete containing by-product wastes like Rice husk ash, cotton stalk, palm leaf ash, and palm oil fuel ash (POFA) has been extensively studied by many researchers [[26], [27], [28], [29], [30]]. These by-product wastes, especially POFA, are rich in silica content and exhibit high pozzolanic characteristics. It has been effectively used as a cement replacement with increased compressive strength and durability [[31], [32], [33], [34]]. Since these materials are available in abundance in east Asian parts of the world, like Malaysia and Indonesia. However, these alternatives are not scalable at the global level [[35], [36], [37], [38], [39], [40]]. Table 4 shows physical and chemical characteristics of superplasticizer.

**Table 2:** Test conditions, SCM replacement levels and cementitious system adopted by various authors

Author	SCM	Replacement levels	Cementitious system/matrix	Admixtures	W/B ratio	Tests conducted
Vance et al. [43]	Fly ash and Metakaolin	5% and 10%	Paste	-	0.35 and 0.38	Compressive strength, Isothermal calorimetry, Thermogravimetric analysis (TGA) and Differential thermal analysis (DTA)
Artelt and Garcia [44]	Fly ash, Limestone and Silica Fume	7% SF in FA cement and 6% SF in LS cement	Mortar	PCP-based superplasticizer	0.22	Spread test, Funnel Flow and Shear test
Danish and Mosaberpanah [11]	Fly ash and Cenosphere (CS)	FA 5%–20% and CS 5%–20%	Mortar	PNS-based superplasticizer	0.35	Workability, Compressive Strength, Flexural strength, Acid attack, Fire resistance and Drying shrinkage
Erdoğdu et al. [45]	Fly ash and Silica Fume	FA 5%, 15% and 20% SF 10%	Concrete	PMS-based superplasticizer	0.51 to 0.65	Compressive strength and Slump loss
Yılmaz and Olgun [46]	Fly ash, Limestone and Dolomitic limestone	FA 5%–40%, LS 5%–15% and DLS 5%–15%	Mortar	–	0.26 to 0.28	Compressive strength, Water demand, Setting time, XRD and SEM
Weerdt et al. [47]	Fly ash and Limestone	5%–35%	Mortar	–	0.5	Compressive Strength, Flexural strength, Thermogravimetric analysis and X-ray diffraction
Uysal et al. [48]	Fly ash, Granulated blast furnace slag, limestone	10%, 20% and 30%	Concrete	PCE- based superplasticizer	0.33	Slump flow, T_50_ time, L-box, V-funnel, Compressive strength and Ultrasonic pulse velocity
Burgos-Montes et al. [49]	Limestone and fly ash	10%, 30% and 50%	Paste	PMS, PNS and PCE-based superplasticizer	0.33 to 0.43	Paste Rheology, Compressive strength and Scanning electron microscope
Zaribaf and Kurtis [50]	Limestone and Metakaolin	10%,20% and 30%	Paste	PCE, LS, PNS and PMS-based superplasticizer	0.40	Mini slump, Thermogravimetric analysis, Isothermal calorimetry, X-ray diffraction.
Antoni et al. [51]	Limestone and Metakaolin	LS 15% and 20%; MK 10%, 20%, 30% and 40%	Mortar and Paste	PNS-based superplasticizer	0.5 and 0.4	Compressive strength, Flexural strength, Thermogravimetric analysis, Mercury intrusion porosimetry (MIP) and X-ray diffraction
Hallal et al. [52]	Limestone and natural pozzolan	20% and 25%	Paste	PNS and PRM based superplasticizer	0.35, 0.40 and 0.45	Marsh cone test
Adjoudj et al. [53]	Limestone powder and Finely blast furnance slag	0–30%	Mortar	PCE-based superplasticizer	0.55	Mini slump test and rheological properties
Li et al. [54]	Metakaolin	0–40%	Paste	HPEG based PCE Superplasticizer	0.5	Mini Slump, Dispersing performance and Adsorption of PCE on Metakaolin blended cement
Li and Ding [55]	Metakaolin and GGBFS	10–30%	Mortar	PCE based superplasticizer	0.44	Fluidity test, Compressive strength and Flexural strength

**Table 3:** Chemical composition and physical properties of different SCMs

Chemical composition	Types of SCMs			
	Fly ash	Limestone	Metakaolin	GGBFS
SiO_2_	54.40–39.50	4.93–0.04	51.40–49.55	40.98–32.71
Al_2_O_3_	16.30–31.01	0.82–0.06	51.70–40.25	15.75–10.82
Fe_2_O_3_	11.05–4.50	0.58–0.05	2.50–0.38	2.5–1.38
CaO	22.77–2.30	56.53–42.3	2.71–0.02	45–34.85
MgO	5.86–1.20	0.93–0.10	1.02–0.08	8.60–6.83
SO_3_	3.56–0.48	–	0–0.99	0.82–0.097
Na_2_O_eq_	2.44–0.34	0.50–0.04	0–0.28	5.42–0.23
LOI	4.30–1.04	43.56–40.40	2.04–1.05	0–0.32
Physical Properties				
SSA (m^2^/kg)	813–247	810–250	15000–11200	630–261
Bulk density (kg/m^3^)	2740–2140	2790–2740	2600–2350	2920–2860
Mean diameter (μm)	2.80–2.15	30.30–10.12	16.80–12.45	–
**References**	[11,13,[55], [56], [57], [58]]	[[47], [48], [49],^51^,[59], [60], [61]]	[[16], [39], [40], [43], [50], [51], [54], [62]]	[48,53,54,56,57,63,64]

**Table 4:** Physical and chemical characteristics of superplasticizer

Author	Type	Solid content (%)	Viscosity (mPa.s)	pH Value	Density (g/cm^3^)	%C	%S	%H	%N	Na (ppm)	K (ppm)
Burgos-Montes et al. [49]	PCE	40.9	118.20	4.5	–	51.67	0.30	8.14	0.17	2820	10
Altun et al. [68]	PCE	48.8	320	3.96	1.14	50.74	–	–	0.34	4500	–
Uysal et al. [48]	PCE	20.5		8	1.04	48.11	–	–	0.15	3640	–
Burgos-Montes et al. [49]	PNS	39.6	51.11	8.5	–	43.78	9.13	4.53	0.80	31,400	340
Burgos-Montes et al. [49]	PMS	41.9	31.50	8	–	18.65	10.65	3.98	22.17	55,280	0.2

**Fig. 3:**
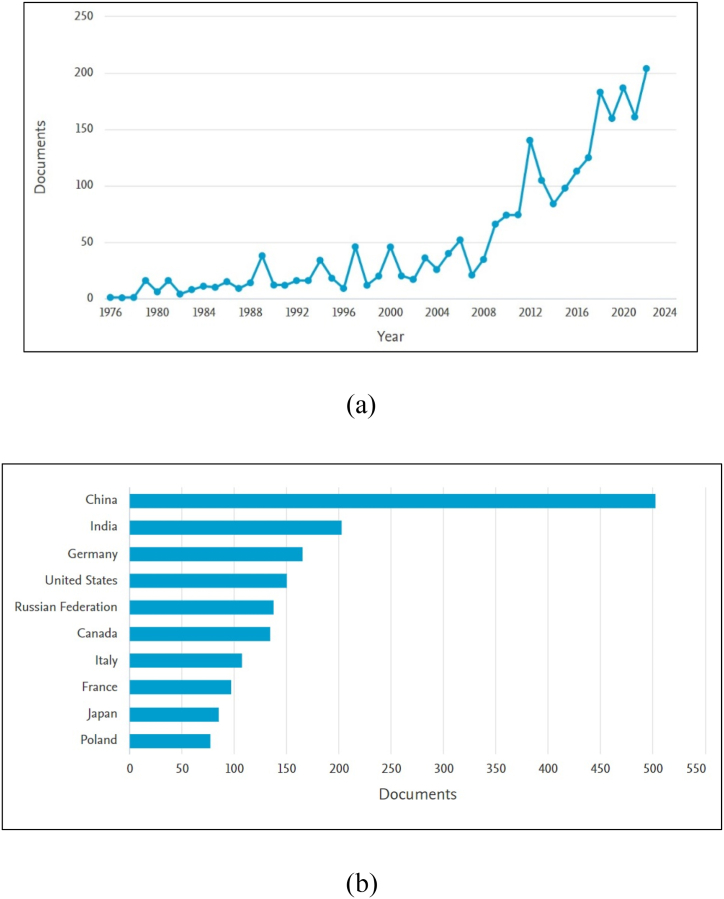
(a) Growth in literature regarding incorporation of high range water reducing admixture in blended cement; (b) Country of origin wise distribution of related literature [42] (Accessed Feb 12, 2023).

**Fig. 4:**
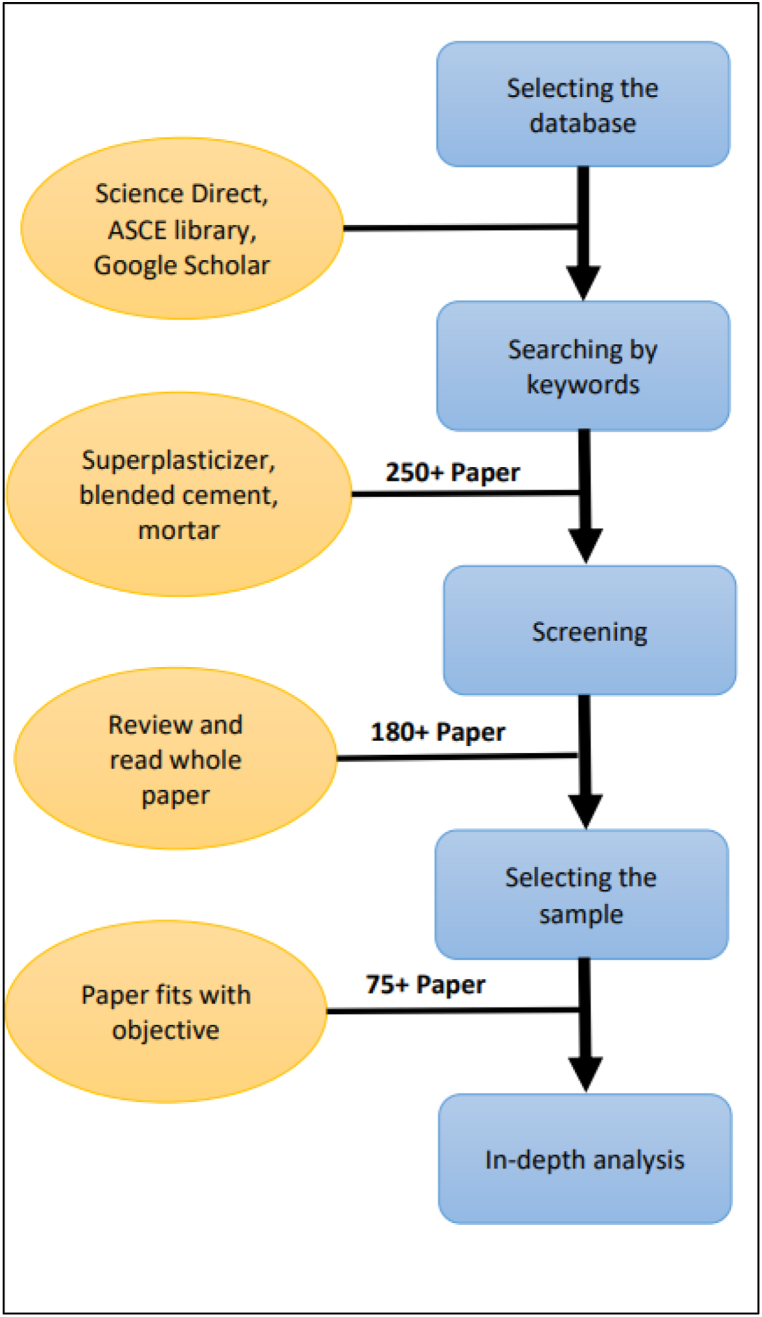
Workflow for the review.

As shown by Anurag et al. [41], there has been an increasing trend in SCM blended cement composite over the past few years. Also, it is evident from Fig. 3 (a), from the year 1976–2022, there has been a sharp increase in work related to superplasticizer in the cementitious system of blended cement. Fig. 3 (b) demonstrates that research is this field is led by China followed by India then Germany and United States. From a sustainability point of view, it is essential to understand the chemical admixture-blended cement interaction. Therefore, the current work intends to assess the influence of high-range water-reducing admixtures especially superplasticizers, on blended cement. Various fresh and hardened properties, along with microstructural and durability studies, were conducted for this review. The literature audited was collected from peer-assessed sources such as Web of Knowledge, Google Scholar, ASCE Library, Science Direct, and many more. Further, papers specifically based on the effect of SCMs with superplasticizers on various physico-mechanical, microstructural, or durability properties were shortlisted, as shown in Fig. 4. By reviewing the research conducted so far, this study contributes to: (i) reducing the knowledge gap that exists in regards to the use of chemical admixtures in low-carbon cement and concrete; (ii) identifying the improvement scope and research gap; and (iii) promoting the utilisation of pozzolanic waste in the construction industry. Also, the recent research activities going on at the author's research institute (CSIR-Central Building Research Institute, Roorkee, India) are also mentioned at the end of this article. The intention of the study was to develop high-strength Limestone Calcined Clay Cement- Silica fume (LC^3^SF) with a target strength comparable to OPC-53 grade. Box-Behnken Design (BBD) was used for multi-objective optimization. Using Design Expert v.11 software, a total of 27 experiments with 3 replications of the central point were designed to allow pure error estimation.

## 2 Chemistry of low carbon cement (LCC) and superplasticizers

### 2.1 Chemistry of superplasticizers

Nowadays, in the construction industry, various types of chemical admixtures are widely used for the numerous benefits that they provide, such as improving pumpability and workability along with providing better durability properties such as freeze-thaw resistance and shrinkage reduction, to name a few. Although the first of these admixtures in concrete is undocumented but ancient Romans, Europeans, and Chinese history suggest using organic materials like juice and latex from rubber plants, polished glutinous rice paste, lacquer, tung oil, and blackstrap molasses in building materials. Lignosulfonate-based, melamine-based and naphthalene-based superplasticizers have been widely used in the concrete industry for a long time, but with the invention of poly-carboxylate comb polymers in 1981, concrete technology got a major breakthrough as this novel class of concrete superplasticizers helped in formulating advance concrete such as self-compacting concrete (SCC), ultra-high strength concrete (UHPC), and provided a longer slump for ready mix concrete [65]. Chemical admixtures are also being used in cold-weather concrete for property enhancement and strength development of cement stabilised materials [66]. These days, superplasticizers are picking up steam due to their cement dispersion ability, high-water reduction effect, and ability to alter polymer structure to achieve desirable properties. Due to the remarkable performance of the currently available superplasticizers, engineers are able to build concrete structures that were unthinkable before. For example, the Burj Khalifa in Dubai, which is the tallest building in the world (830 m), used Vinyl ether-based polycarboxylate ether (PCE) and retarders. The concrete was pumpable up to 650 m and had a retention time of 3 h at a temperature of 50 °C. Although studies have also shown that in case of concrete, PCE polymer showed retardation of early cement hydration and thus slacks up early strength development. Yang et al. [67] showed that Methacrylate ester-based PCE admixture can also be used as an effective grinding aid during the milling of cement clinker, exhibiting the versatility of PCE-based admixture application in the construction industry.

#### 2.1.1 Types of PCEs

1. Methacrylate ester-based (MPEG) PCEs
2. Allyl ether-based (APEG) PCEs
3. Vinyl ether-based (VPEG) PCEs
4. Methallyl (C4) ether-based (HPEG) PCEs
5. Isoprenol (C5) vinyl ether-based (IPEG) PCEs
6. Zwitterionic (amphoteric) PCEs
7. Organo-silane modified PCEs
8. Cross-linked PCE molecules (XPEG-type PCEs)
9. Phosphated PCEs

Cement particles have a tendency to agglomerate and flocculate, which is dependent upon factors such as particle size, nature, and composition. The correlation between solids content and viscosity of OPC is given by following equation called ‘Krieger-Dougherty’ equation.

$$\frac{\eta }{\eta_{C}}={\left(1-\frac{\emptyset }{\emptyset_{m}}\right)}^{-\left[\eta \right]\emptyset_{m}}$$

**Fig. 5:**
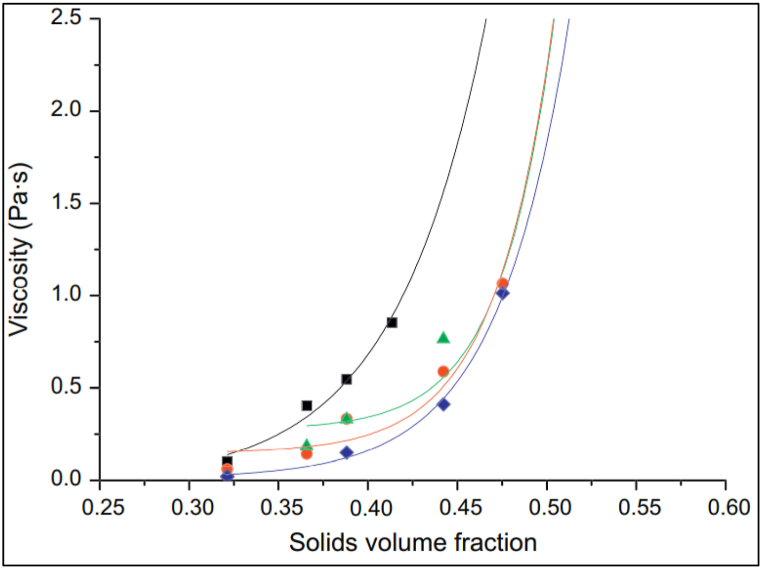
Viscosity vs solid volume fraction for Portland cement paste with and without admixtures (black line represents paste without admixture and green, orange and blue line represent paste with PMS, PNS and PCE superplasticizers respectively [49].

**Fig. 6:**
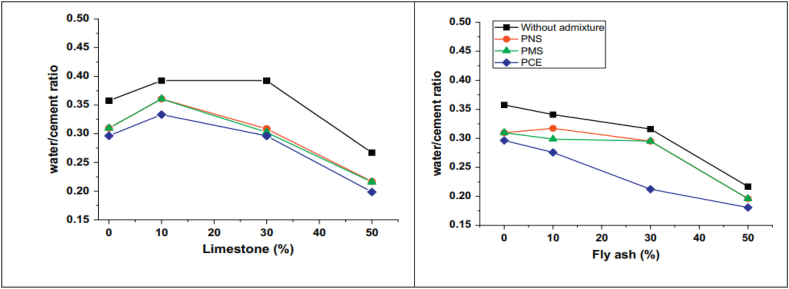
Minimum water-binder ratio required to achieve maximum viscosity of blended cement paste [49].

Where η is viscosity, $\eta_{C}$ is viscosity in continuous phase, $\emptyset_{m}$ is maximum solids content by volume and [η] is intrinsic viscosity. For a given ordinary portland cement paste without superplasticizer, as the maximum solid content reached from 0.40 to 0.45, the viscosity showed an exponential rise (Fig. 5). When superplasticizer was added, a much lower water-binder ratio could be achieved as maximum solid content increased. In the case of blended cement, the water-binder ratio required to achieve maximum viscosity, varies with varying replacement levels of SCMs (limestone and fly ash), as shown in Fig. 6. From the figure, it can be observed that, for limestone blended cement as replacement increases from 0% to 10%, the water/cement ratio also rises, but with further replacement, the ratio sharply decreases. Whereas for fly ash blended cement, the water/cement ratio only decreases with increasing replacement level. Thus, the addition of superplasticizer to ordinary portland cement and blended cement do actually reduces the amount of water required to obtain the same workability.

**Fig. 7:**
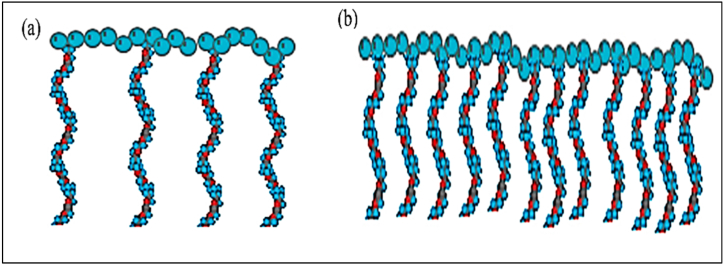
Structural representation of PCE-type superplasticizer, (a)type used in precast for early strength (carboxylate to side chain ratio of 2–3:1) and (b) type used in ready-mix concrete for slump retention (carboxylate to side chain ration of 6:1) [70].

**Fig. 8:**
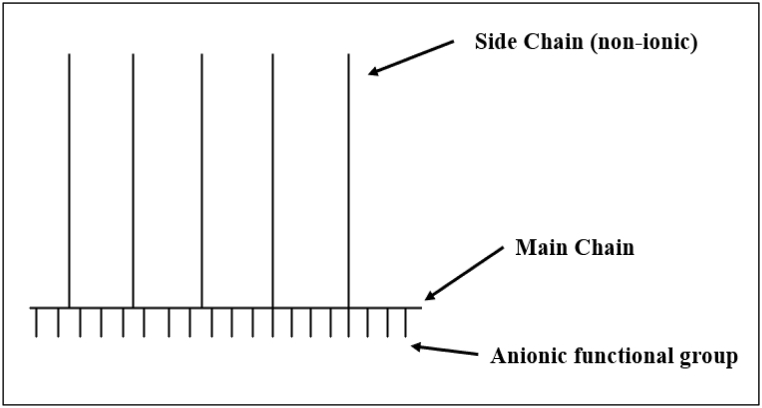
Chemical structure of Water reducing admixture [71].

**Fig. 9:**
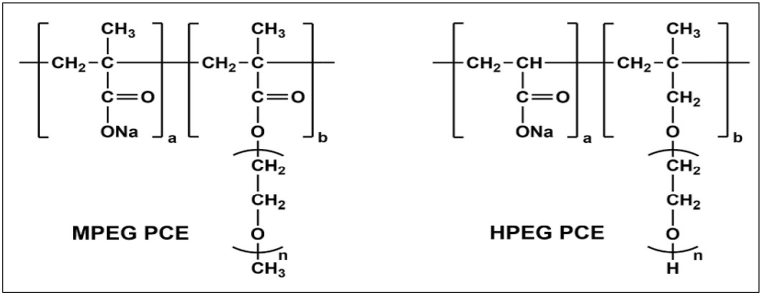
General Molecular structure of MPEG-PCE and HPEG-PCE; (a:b = 2–20:1, n = 25,45 and 114) [74].

The structure of PCE-based superplasticizers has a comb-like structure that consists of a negatively charged polyethylene main chain, hydrophilic polyethylene glycol side chains, and carboxylate functional groups as indicated in Fig. 7, Fig. 8 [[69], [70], [71]]. The superplasticizer prevents flocculation by adsorbing on the cement grain surface and also reduces the entrapment of water in the flocs, thus significantly reducing the water demand to obtain the same fluidity in the cement matrix. The synergy between the carboxylic group and polyethylene oxide/polypropylene oxide side chains determines the efficiency of superplasticizer in cementitious systems. Winnefeld et al. [72] and Zingg et al. [73] observed that admixture adsorption played a crucial role in functioning of PCE-based admixture and was influenced by side chain length, molecular weight and charge density, as shown in Fig. 9.

**Fig. 10:**
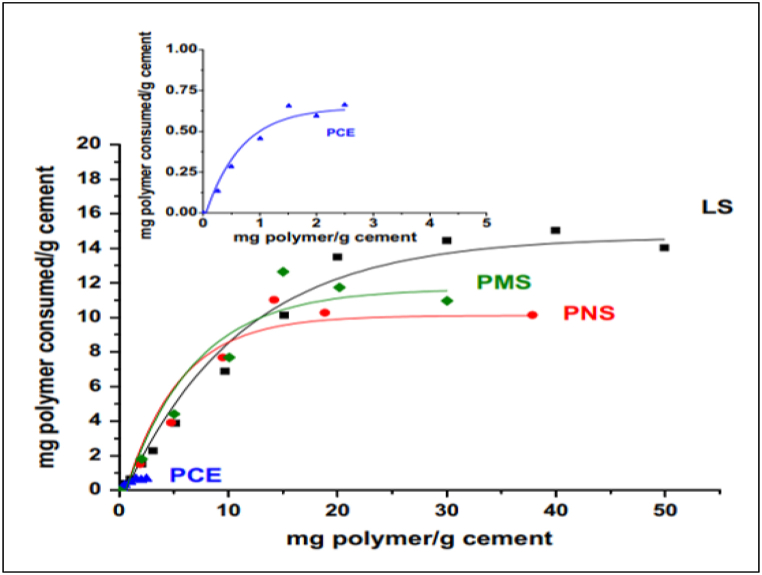
Adsorption isotherms of different superplasticizers [64].

As PCE-based admixture dosage increased, the adsorption of admixture on cement particles also increased due to the inter-polymer crosslinking effect of Ca^2+^ ions. This effect was also highlighted by Burgos-Montes et al. [64] in their work. Fig. 10 compares the adsorption of different types of admixtures onto cement particles. The authors opined that admixture adsorption was linear for low concentration of admixture, which converts to plateau adsorption as concentration of admixture increased. It indicated that admixture was not being adsorbed by cement. It can also be said that adsorption capacity depends on various factors of the polymer, such as main chain length, density, side chain length, etc.

For the same adsorption, PCE-based admixture requires a lesser dosage as the polymer has a larger radius of gyration and, thus, forms a thicker layer compared to other polymers. It is also worth noting that alkali content of cement affects the compatibility of cement with superplasticizer [74,75].

**Fig. 11:**
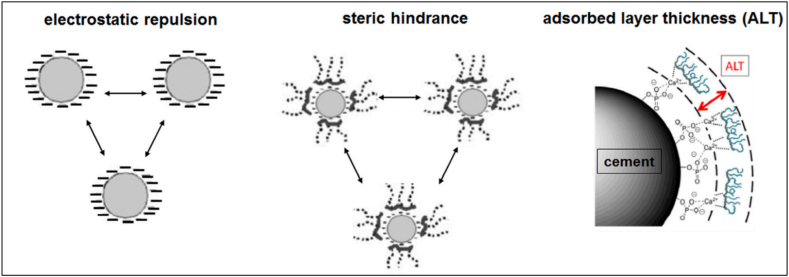
Representation of electrostatic repulsion and steric hindrance mechanism of superplasticizer [70].

**Fig. 12:**
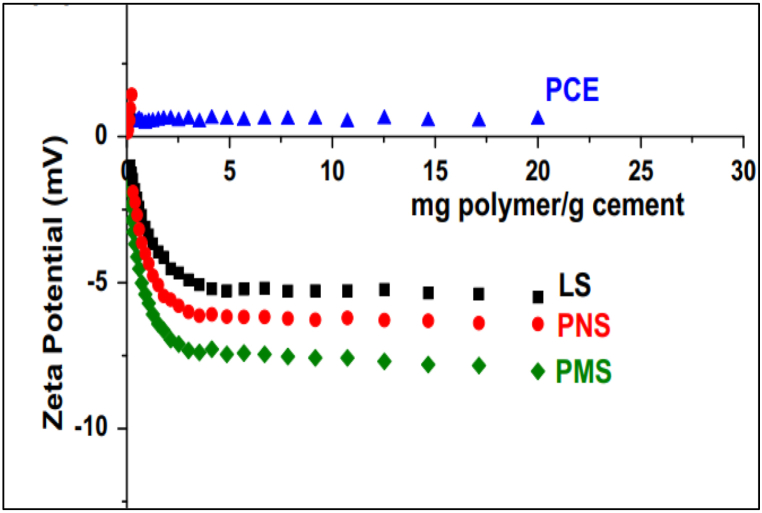
Variation of Zeta Potential on Ordinary Portland cement [64].

In traditional admixtures such as Polymelamine-based (PMS), Polynaphthalene-based (PNS), and lignosulfonate-based (LS), dispersion of cement particles happens by an electrosteric mechanism supporting repulsion and hindering flocculation (Fig. 11). Whereas in the case of PCE-based admixture, it creates a steric hindrance to inter-particle connection. Zeta potential (ζ-potential) helps in understanding the surface charge of particles and the effect of electrostatic dispersion forces between polymer and particle. To effectively quantify the efficiency of a superplasticizer, it is important to understand how much polymer is interacting and how much is left over on the particle. ζ – potential measures the voltage difference between surface of a suspended particle and the electrolyte solution in which it is submerged. To study the cement-superplasticizer interaction, the change in particle charge is observed as the superplasticizer concentration changes. OPC inherently has a slightly positive zeta potential, thus making it easier for anion group superplasticizers to absorb. PCE based admixture has a zeta potential nearly zero indicating electrosteric mechanism of repulsion contributes negligible; instead, PCE based admixture induces a steric obstacle mechanism for repulsion. As it is evident from Fig. 12, the PMS has the highest electrical contribution, and the PCE has a zeta potential of approximately zero. It was also observed by Fares et al. [76] that inter-floc interaction known as yield stress also reduced in cement paste by as much as 90%. The physical and chemical properties of cement and the adsorption of superplasticizer change in the presence of mineral admixtures. Thus, the behaviour of the cement matrix is greatly influenced by the main chain group, side chain group, functional group, and zeta potential of the superplasticizer used. The sterical mechanism used by PCE-based superplasticizers stabilises cement particles at much lower dosages.

### 2.2 Effect of blended cement incorporating superplasticizers

**Fig. 13:**
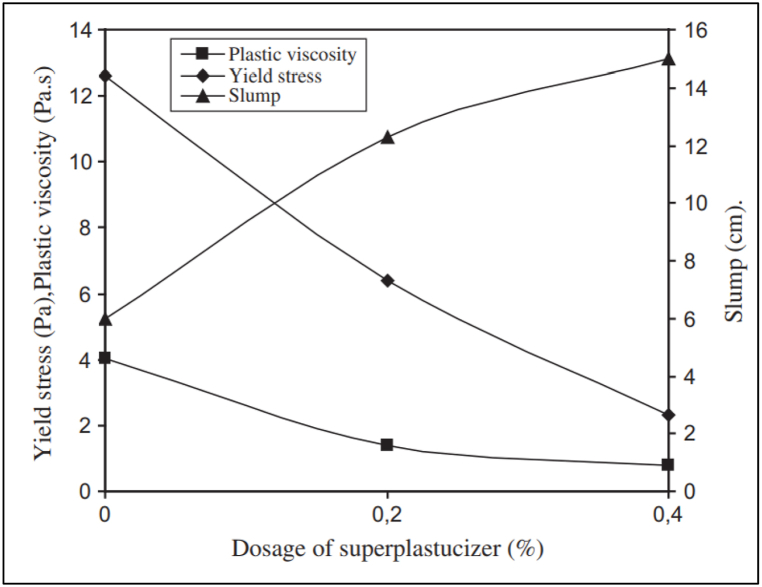
Variation of rheological properties of Portland cement mortar with respect to superplasticizer dosage [53].

**Fig. 14:**
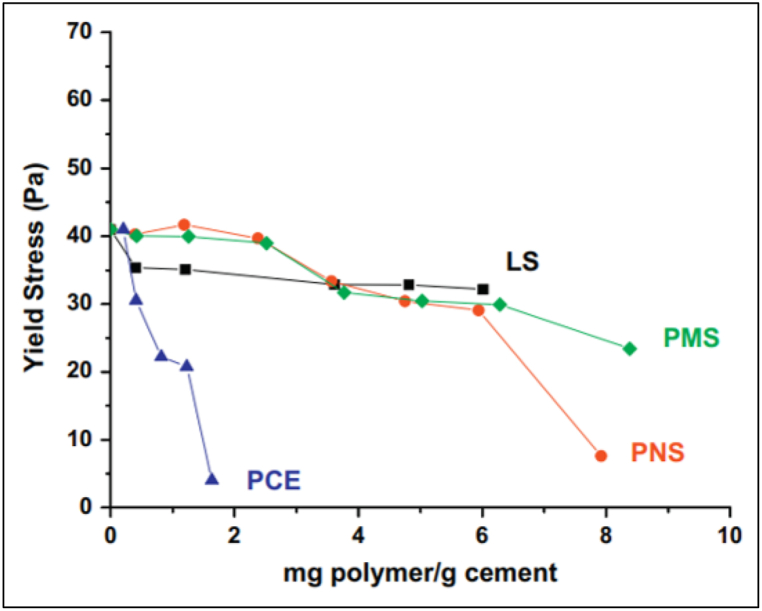
Yield Stress variation in ordinary Portland cement paste [63].

In cementitious matrix, the efficiency of dispersion phenomena depends on the absorption of superplasticizer, which further depends on the surface charge of binder particles. The extent and effect of superplasticizer absorption on OPC and blended cement is a function of properties such as surface charge. Yield stress is the minimum stress required to deform a cementitious matrix, whereas plastic viscosity is the internal resistance offered by the cementitious matrix towards deformation. Fig. 13 shows the rheological properties like yield stress, plastic viscosity, and slump of OPC mortar with superplasticizer, whereas Fig. 14 shows the yield stress variation of OPC with various superplasticizers. With the addition of FA, the superplasticizer dosage can be reduced. As the work of Hallal et al. [52] showed, when 12% of fly ash was added to cement, the superplasticizer requirement decreased by around 35%. Adsorption of LS-based superplasticizer on cement containing FA was lower than that of non-blended cement, whereas for PMS and PCE, adsorption was greater. Fly ash particles had a zeta potential of +2.5 mV, which favoured polymer adsorption. Fly ash blended cement paste with PMS and PNS admixtures showed decline of 75% in yield stress thus behaving similar to non-blended cement rheologically. Burgos-Montes et al. [64] added 30% limestone to cement paste and concluded that the adsorption of PCE and PNS was the same as compared to non-blended cement, whereas for LS and PMS; the adsorption was higher. This behaviour corresponding to limestone can be attributed to the difference in surface adsorption rate (mg of admixture per m^2^ of cement).

The limestone particles have a high zeta potential (+14 mV), which facilitates the adsorption of the traditional admixture as their zeta potential is over −20 mV. In terms of yield stress, limestone blended cement showed a decline in yield stress (<1 Pa), resulting in better paste rheological properties, as also shown by Adjoudj et al. [53]. For FA and LS blended cement, it can be said that these SCMs increase the absorption rate and decrease the yield stress of the cementitious matrix.

Strength activity index (SAI) is a method used to assess the quality of SCM blended in cement. Burgos-Montes et al. [49] evaluated the strength activity index of limestone and fly ash blended cement with the addition of PCE-based superplasticizer and found a SAI of less than 1. This indicated no advantageous effect on strength even though a lower water-binder ratio was used. The authors explained the reason for low reactivity as, SCMs persuades dilution effect and large proportion of SCMs remained unreactive.

The interaction of a superplasticizer with any clay mineral is governed by two mechanisms: surface absorption and chemical intercalation. In surface absorption, the anionic superplasticizer polymer gets absorbed onto a positively charged aluminosilicate sheet of clay minerals [53,74]. While in the intercalation mechanism, the polyethylene glycol side chain gets coupled with silanol present in the interlayer gallery of aluminosilicate sheets, thus trapping the superplasticizer. Thus, the available superplasticizer for dispersion decreases.

**Fig. 15:**
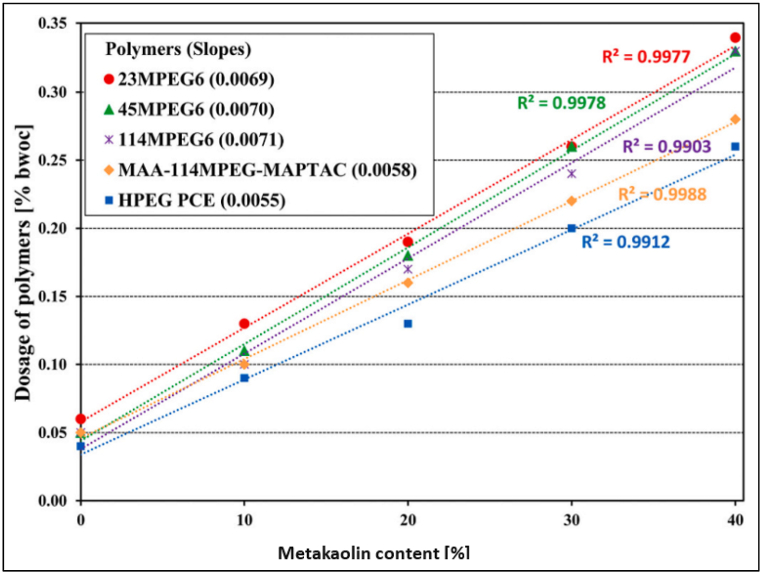
Dosage of superplasticizer required for metakaolin blended cement (126 ± 0.5 mm of flow) [54].

Metakaolin blended cement has better fluidity because superplasticizer absorption is high. This was due to release of calcium ion (by cement dissolution as pore solution), which gives positive charge when absorbed onto metakaolin particles [53]. But as the replacement level of metakaolin rises, the superplasticizer dosage also increases, as fewer calcium ions are present to activate the metakaolin particle, resulting in less absorption. As concluded in the study by Li et al. [54], as the replacement level increased to 40%, the superplasticizer dosage increased 6 times (Fig. 15). The authors were skeptical about the absorption of superplasticizer on metakaolin particles as the surface charge of metakaolin was highly negative, i.e., −30 mV. It was assumed that, as per the DLVO theory (developed by Derjaguin, Landau, Verwy, and Overbeek), particles with such a high negative charge would show strong electrostatic repulsion, but it was found that metakaolin particles exhibited poor dispersion due to high slurry viscosity. Consequently, PCE-based superplasticizers, which are effective in neat OPC, will also be effective in metakaolin blended cement. Out of HPEG and MPEG-based PCE structures, the former polymer is more effective in dispersion performance.

**Fig. 16:**
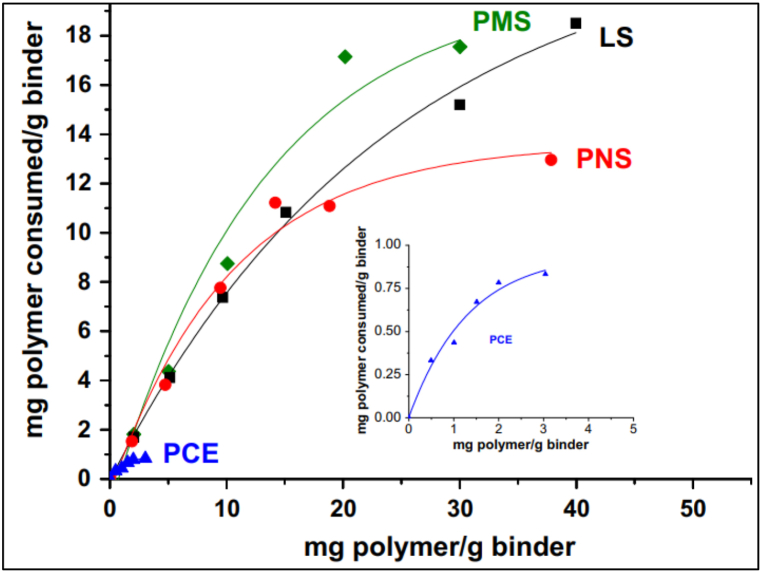
Superplasticizer adsorption isotherms of Silica Fume blended cement [64].

In the case of silica fume blended cement, interaction between cement and silica fume particle led to higher consumption of admixtures. This can also be seen in adsorption isotherms (Fig. 16), as no adsorption plateau was observed due to the large specific surface area of SF. In order to achieve the same decline in yield stress (as fly ash blended cement), higher doses of admixture were required for SF blended cement. So, it was concluded that rheological behaviour of SF blended cement paste is affected by physical and chemical properties of SF particle due to which water and superplasticizer demand rises [44,45,77,78]. Artelt and Garcia [44] also pointed out in their work that the addition of SF increased the surface area in the cement matrix enormously, thus reducing the amount of admixture available per unit surface area. Due to this effect, steric particle interaction was also reduced.

When GGBFS is replaced with cement in mortar without superplasticizer, the yield stress tends to increase and plastic viscosity tends to decrease with replacement rate. Although this opposite behaviour also depends on the particle size distribution of GGBFS. The limestone-metakaolin blended cement without superplasticizer showed a decrease in fluidity as the replacement level increased. When LS was added to MK in cement matrix, the thixotropy increased due to high specific surface area and platelet like structure of metakaolin which traps water in-between interlayers. Thus, it can be said that the specific surfaces of SCMs play a very important role in admixture adsorption and dosage.

## 3 Hydration mechanism

There are two mechanisms of hydration of cement: one is through-solution hydration, and the other is solid-state hydration. It is believed that during the initial phase of hydration, the through-solution mechanism is dominant and that later, the solid-state mechanism swings into action. The hydration of Portland cement is a well-established process. The aluminates hydration starts first and is responsible for the loss of consistency and solidification of the paste. While silicates provide paste with hardening characteristics [79].

**Fig. 17:**
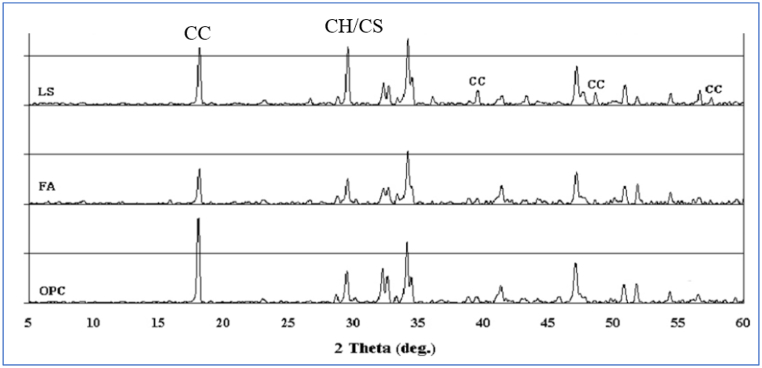
XRD pattern of Portland cement and FA blended and LS blended cement cured at 2 days [46].

Portland cement clinker contains Bogue's compound, i.e., tricalcium aluminate C_3_A (Ca_3_Al_2_O_6_), tetra-calcium aluminoferrite C_4_AF (Ca_4_Al_n_Fe_2-n_O_7_), tricalcium silicate C_3_S (CaSiO_5_), and dicalcium silicate C_2_S (Ca_2_SiO_4_), as shown in equations (1), (2), (3), (4)). The hydration of the clinker phase results in the formation of portlandite, C–S–H, ettringite, and AFm phases. Fig. 17 shows the presence of calcium hydroxide (CH) in OPC paste.

(1) $$2C_{3}S+11H\;\to \;C_{3}S_{2}H_{8}+3\mathrm{CH}$$

(2) $$2C_{2}S+9H\;\to \;C_{3}S_{2}H_{8}+CH$$

(3) C3A+3CSH2+26H→C6AS3H32

(4) C4AF+3CSH2+21H→C6(A,F)S3H32+(F,A)H3

Generally, SCMs demonstrate slow reactivity. The reactivity of SCMs depends on factors such as fineness, chemical composition, and reactive phases present. SCMs, along with providing silica, also provide reactive alumina, which, when reacts with calcium hydroxide and sulfate ions results in the formation of AF_t_, AF_m_ and C_4_AH_13_.

Blending fly ash increases C–S–H formed at lower Ca/Si ratio along with amount of AF_m_ phases. Fly ash, being pozzolanic material, remains non-reactive until the development of calcium hydroxide from the cement reaction in the system [57]. For fly ash containing CaO more than 20%, Papadakis [56] suggests that due to the synchronous pozzolanic and cementitious reactions of FA with cement, the hydration is much more complicated as compared to other blended cements. In the case of high calcium fly ash, which contains nearly 40% SiO_2_, the pozzolanic reactivity of this pure silica results in the formation of calcium silicate hydrate (C–S–H), as represented by equation (5).

(5) $$2S+3CH\;\to C_{3}S_{2}H_{3}$$

The author also attributed the high early strength due to the presence of tricalcium aluminate (C_3_A) and monocalcium aluminosulfate (C_4_A_3_ $\bar{S}$). During hydration reaction alumina products such as C_4_AH_13_, C_4_A $\bar{S}$ H_12_, C_3_A.3C $\bar{S}$.H_32_ and C_3_AH_6_ result in formation of ettringite and tricalcium aluminate hydrate which eventually convert into Ca-monosulfoaluminate and tetracalcium aluminate hydrates as shown in equations (6), (7)).

(6) $$A+C\bar{S}\;H_{2}+3CH+7H\;\to \;C_{4}A\;\bar{S}\;H_{12}$$

(7) $$A+4CH+9H\;\to \;C_{4}{AH}_{13}$$

In the case of LS blended cement, Fig. 17 shows a strong peak of calcium carbonate (CaCO_3_ also called CC) [46]. The reason for this CC was, as unreacted LS or reaction between Ca(OH)_2_ and C–S–H. At 7 days, the crystalline phase formed was enttringite (Af_t_) and some unreacted di- and tricalcium silicate, which decreased as curing time increased (Equations (8)–(10)).

(8) $$C_{3}A+3{CaSO}_{4}.2H_{2}O+H_{2}O\;\to \;C_{3}A.3{CaSO}_{4.}32H_{2}O$$

(9) $$C_{3}A.3{CaSO}_{4.}32H_{2}O+H_{2}O\;\to 3C_{3}A.{CaSO}_{4.}12H_{2}O$$

(10) $$3C_{3}A.{CaSO}_{4.}12H_{2}O+2{CaCO}_{3}+18\;H_{2}O\;\to \;2C_{3}A.{CaCO}_{3.}11H_{2}O+C_{3}A.3{CaSO}_{4.}32H_{2}O+3CaO.{Al}_{2}O_{3}.0.5Ca{\left(OH\right)}_{2}.0.5{CaCO}_{3.}11.5H_{2}O$$

The reason for replacing OPC with MK was the aluminous complexion of MK, which helps in the formation of carboaluminate phases. The MK (Al_2_O_3_·2SiO_2_) formed from the calcination of clay reacts with portlandite (CH) from cement hydration, resulting in the formation of C–S–H gel along with calcium aluminate hydrate (C_4_AH_13_) and alumino-silicate hydrates [80]. GGBFS in itself contains cementitious properties; thus, calcium hydroxide is not required to form C–S–H. But this cementing property is not sufficient to use GGBFS as a stand-alone material; therefore, it is blended with Portland cement. Due to the presence of calcium hydroxide and gypsum, the hydration process is accelerated [54].

## 4 Fresh properties

### 4.1 Workability

**Fig. 18:**
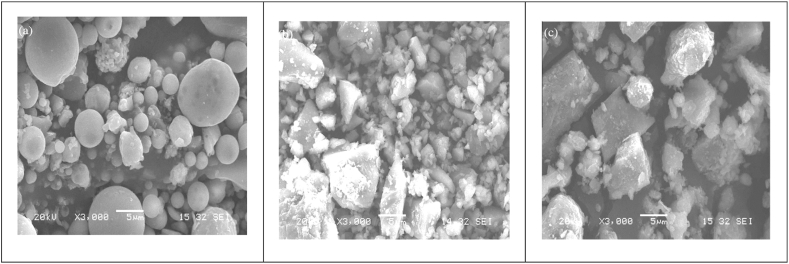
Secondary electron images of (a) FA particle having smooth surface texture, (b) and (c) GBFS and Limestone particle having rough and angular texture respectively [48].

Workability of mortar/concrete is dependent upon the dosage of superplasticizer and the type of blended cement used. It is well understood that with the introduction of PCE-based superplasticizer in cementitious system, steric hindrance and electrostatic repulsion avoids flocculation and thus the fluidity of cementitious system is vastly enhanced. To ease the injectability and penetrability of the grout mixture used for filling post-tensioning ducts, anchorage sealing and injection, grouting with high flowability are required. Chandra and Björnström [81] studied the effect of different superplasticizers on Portland cement. The authors found that the type of cement greatly influenced the adsorption of superplasticizer, thus affecting the workability. The workability of cement paste depends on the dispersion ability of the superplasticizer and the amount of superplasticizer adsorbed on the paste. The adsorption was not linear for all the compounds within ordinary portland cement, either. For example, superplasticizer adsorption on alite and belite was less compared to adsorption on C_3_A and C_4_AF [52]. It was found that the workability of mortar significantly increased with PCE dosages beyond 0.2% [53]. The effect of PNS was observed to be the weakest amongst all admixtures. Authors also reported that for PNS, with an increase in dosage, there was a significant increase in fluidity. Considering the structure of superplasticizer, Özen et al. [71] studied the effect of having different side chain lengths on fresh properties of cement paste and mortar. It was found that admixtures with too long or too short side chain adversely affected the workability of mortar due to weak steric hindrance. A side chain molecular weight of 2450 g/mol was found to be optimal for the best workability of cementitious systems. Alaka and Oyedele [58] worked on exceptionally low water binder ratio for high fly ash replacement of cement with the help of surplus dose of PCE-based superplasticizer. The authors found that to achieve a target slump of 50 mm–90 mm, the minimum water/binder ratio was 0.35. This shows that as the superplasticizer dosage increased, water demand was also increased for fly ash blended concrete matrix. It was also observed that the fly ash with a higher loss of ignition (LOI) demands a higher water-to-binder ratio. It was generally agreed that the workability of cementitious system decreased due to a larger surface area and finer particle size, but the long-term permeability improved [48]. However, workability of mortar or concrete is also influenced by the shape of SCM particles, particle surface smoothness, and particle size distribution, as evident from Fig. 18. In the study conducted by Danish and Mosaberpanah [11], with 20% FA, the fluidity was increased due to the fact that the particles of FA were spherical in shape, thus acting as ball bearings and providing a lubricant effect. This increase in fluidity was also observed due to a 23% reduction in packing density (reduced to 0.63) as compared to the control mix. Even with 5% cenosphere and 15% FA, workability was increased by 16%–18% as compared with the control mix. This improvement in fluidity with the addition of cenosphere was ascribed to the filler effect and nearly perfect spherical shape of cenosphere, which forms a layer of water around its surface, thus working as a ball bearing. Uysal et al. [48] also concluded that, in the presence of superplasticizers, partial replacement of fly ash up to 30% gives the highest workability as paste density is reduced, hence paste volume increases, and friction between fine aggregate and cement paste is reduced, resulting in improved cohesiveness and plasticity. The increase in fluidity and workability was due to the spherical shape of FA particles, which leads to a reduction of inter-particle friction in the concrete mix. In terms of time required to reach 50 cm slump-flow (T_50_) value (concrete with 15% fly ash replacement), the value lies in range of 2–5 s suggesting excellent workability for self-compacting concrete.

**Fig. 19:**
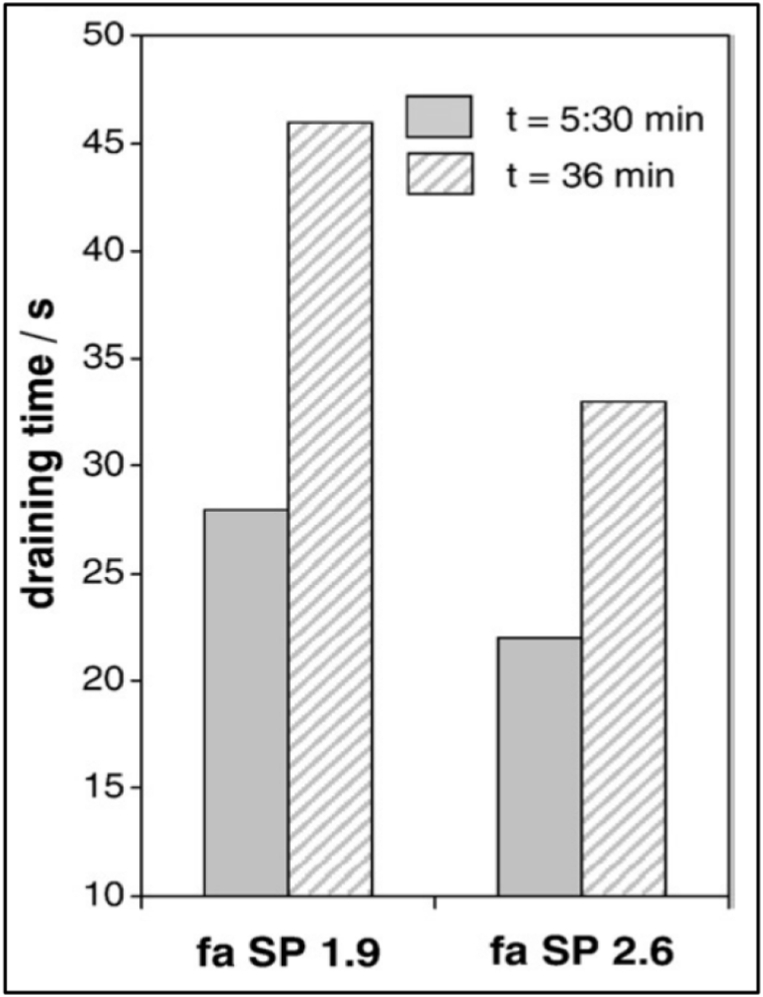
Flow value results for varying HRWRA dosage [44].

Artelt and Garcia [44] studied the rheological behaviour of mortar and showed that the draining time decreased with an increase in superplasticizer dosage, indicating a lower mortar viscosity (Fig. 19). The authors attributed this occurrence to the hydration of C_3_A phase, which requires more water and produces ettringite on the cement surface. However, in the case of concrete, with an increase in FA replacement, the slump loss increases as time elapses, as found in a study conducted by Erdoğdu et al. [45].

Zaribaf and Kurtis [50], in their study on LS blended cement indicated that PMS and PCE-based superplasticizer both produced same workability whereas, LS and PNS-based superplasticizer required higher dosage to achieve similar workability. Although Hallal et al. [52], using Marsh cone test, opined that cement with LS provided better fluidity with 0.8% of PNS-based superplasticizer. Akhlaghi et al. [82] reported that LS had more affinity towards superplasticizer thus optimum dosage was also higher to achieve same workability. Due to particle geometry, surface chemistry and size of particle, water uptake capacity of MK was higher compared to Portland cement [83]. When MK was added, the workability usually decreased [54,84]. As MK replacement level was increased in cement, the superplasticizer dosage required to achieve desired workability also increased linearly. Zaribaf and Kurtis [50], concluded that PCE-based superplasticizer was most effective to improve workability due to its dispersion mechanism without changing hydration kinetics of paste. In particular, HPEG PCE-based superplasticizer are more efficient compared to MPEG PCE-based superplasticizer as the former required half the dosage of latter to achieve same workability [53,74]. Akhlaghi et al. [82] reported that with increase in PCE-based superplasticizer from 0.3 wt% to 1 wt% (of binder); the workability increased, and then it reduced as excess PCEs cause bridging flocculation. Blending GGBFS with wide particle size distribution improved the flow properties of blended cement as small GGBFS particles fill between cement particles [53]. Generally, with addition of 15%–30% replacement of cement with GGBFS, in presence of PCE-based superplasticizer, the workability was found to improve significantly [85]. As compared to Portland cement, SF blended cement concrete does not possess good workability because particle size of SF was very small compared to cement particle which resulted in larger surface area which required more water to fulfil hydration reaction. Workability which is defined as ‘the ease with which freshly prepared cement/concrete mix can be placed’, is influenced by factors like compounds present in cement, shape and size of SCMs, chain length of superplasticizer used and more. For FA, replacement up to 15% without superplasticizer improves the workability and with superplasticizer replacement level up to 30% showed improved workability. In case of LS and MK blended cement; the admixture dosage is increased comparatively due to their better affinity.

### 4.2 Slump retention

**Fig. 20:**
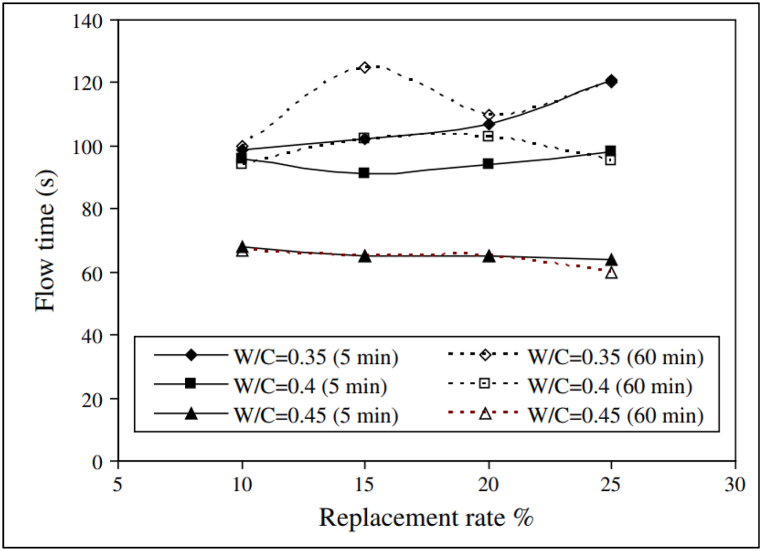
Change in flow time with replacement level of limestone [52].

The loss of workability of mortar or concrete with time is known as slump loss. The addition of a superplasticizer helps in obtaining a high level of workability for a longer period of time. Slump retention mechanism by superplasticizer is due to the continuous release of carboxylate group from hydrolysis of ester, which takes place in alkaline condition of cement pore solution. Saric-Coric et al. [86] studied the influence of PNS and PMS-based superplasticizers on cement paste and found that slump loss depends on the type of superplasticizer. Paste containing PNS-based superplasticizer showed a lower loss in mini slump as compared to paste prepared with PMS-based superplasticizer. The dispersant efficiency of PNS-based superplasticizer was better than PMS-based superplasticizer. Similarly, Hallal et al. [52] showed that LS blended cement has less fluidity loss with PNS-based superplasticizer. The authors also establish the fact that fluidity loss was also influenced by the replacement level of LS in cement, as evident from Fig. 20.

**Fig. 21:**
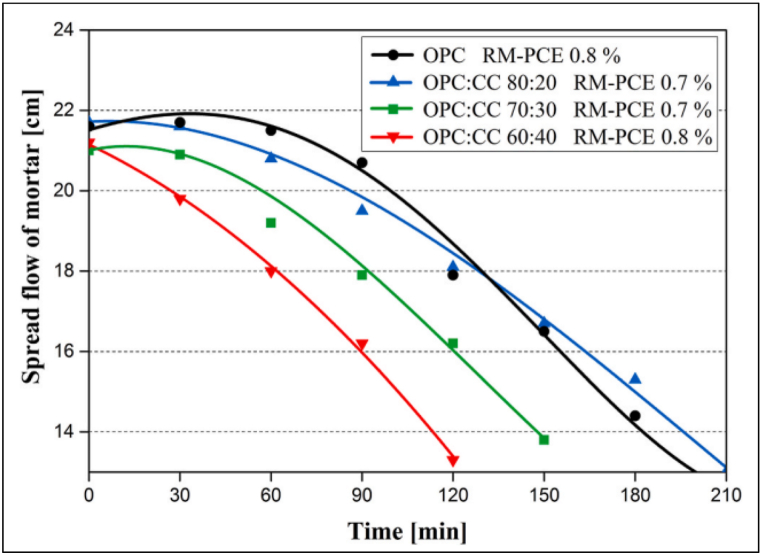
Slump retention of metakaolin blended cement with PCE-based superplasticizer. CC represents calcined clay [1].

In the case of metakaolin blended cement, slump loss was quite fast, which is the major limitation of MK blended cement. In the work conducted by Li et al. [1], OPC with PCE-based superplasticizer exhibited good slump retention up to 90 min. But with MK blended cement as a replacement level increased the retention time decreased. For example, with 20% replacement the retention time decreased to 60 min. Fig. 21 shows the decrease in retention time for different replacement levels of MK in OPC.

As opined by Akhlaghi et al. [82], PCE-based superplasticizer performs better in slump retention as compared to other admixtures in limestone-metakaolin blended cement. PCE-based superplasticizer with lignosulfonate has shown some promising results with slump retention up to 3 h for limestone-metakaolin blended cementitious systems [87].

### 4.3 Setting time

The solidification of the cement matrix due to the hydration reaction of predominantly aluminates determines the setting time. The presence of PCE-based superplasticizer is generally believed to retard the hydration mechanism of cement. The concentration of Ca^2+^ ions, which are present in the main chain carboxylate group, was strongly related to the deceleration of the hydration mechanism. Zhang et al. [60], studied the concentration of Ca^2+^ ions by titration of PCE-based superplasticizer in Ca(NO_3_)_2_ solution (20 mmol/L, pH of 11). The authors concluded that Ca^2+^ concentration drastically decreased as PCE-based superplasticizer was added to solution suggesting a strong synergy between PCE molecules and Ca^2+^ ions.

The addition of MK to Portland cement improved the cement hydration mechanism and reduced the setting time. Aluminium oxide and silicon dioxide in MK quickly react with hydrates of cement and form C–S–H gel. In a study conducted by Li and Ding [55], in which MK was substituted by 10% (with 1.6% PNS-based superplasticizer), the initial setting time decreased by 26% and the final setting time decreased by 36%.

GGBFS blended cement needs calcium hydroxide from OPC to start the hydration, and thus the setting time was increased. With GGBFS substitution up to 30%, the initial setting time was increased by 44%, and the final setting time was increased by 81% [53]. It can be said that setting time, which is directly related to the hydration of aluminates, is a function of the type of SCMs and admixture used in the matrix. But with the addition of superplasticizer, the setting generally retards.

### 4.4 Isothermal calorimetry

In a typical heat of hydration curve of OPC cement, the first peak, which lasts a few minutes, represents alite hydration and the heat generated by the solution of aluminates and sulfates. After 4–8 h s peak is observed which indicates formation of ettringite and also heat of formation of C–S–H. Zaribaf and Kurtis [50], found that with 30% metakaolin replacement; superplasticizer tends to delay the hydration of calcium silicate and aluminate phases as indicated by delay in peak formation up to 4 h. In case of PCE-based and PNS-based superplasticizer, the hydration in terms of calcium silicate and aluminate got delayed due to enhanced dispersion. But in the case of 30% metakaolin replacement and an LS-based superplasticizer, the peak was developed 5 h earlier than the control because of the very early formation of ettringite and the accelerated hydration of C_3_A. It has also been observed in previous studies [88,89] that the absorption of LS-based superplasticizers is significantly high.

**Fig. 22:**
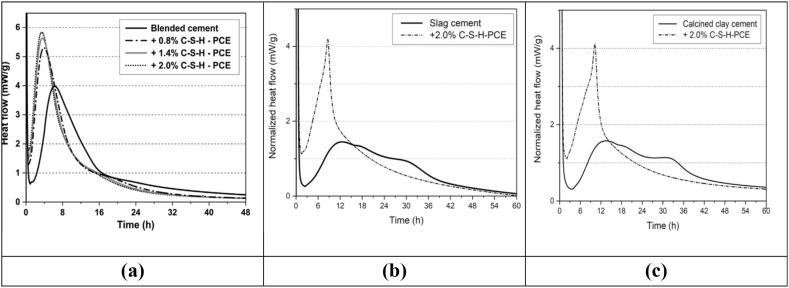
Heat flow and cumulative heat of (a) fly ash (b) GGBFS and (c) MK added to cement paste containing a different proportion of C–S–H–PCE nanocomposite superplasticizer [90,91].

The effect of PCE-based superplasticizer on the hydration rate of the MK, FA, and GGBFS incorporated cement paste was analysed using isothermal calorimetry. Generally, the maximum rate of heat flow increased with an increase in PCE, which was observed due to the growth of portlandite and C–S–H phases. The maximum cumulative heat flow was found at 2.0 wt% addition of PCE-based superplasticizer in FA blended cement, as shown in Fig. 22 (a). This shows that hydration of silicate phases (C_3_S and C_2_S) takes place much earlier and thus accelerates cement hydration significantly. Similar behaviour of PCE based admixture with MK and GGBFS was also observed (see Fig. 22 (b) and (c)), where hydration of silicate phases is accelerated and total heat released also increases following crystallization of C–S–H and portlandite phases.

Apart from this, PCE-based superplasticizer also activated the pozzolanic reactions of the MK and GGBFS, resulting in the formation of secondary C–S–H in the hardened cement [90,91]. Xie et al. [57] investigated the effect of FA on the heat released from cement paste. It was observed that PCE-based superplasticizer accelerated the hydration rate of cement paste. Further, common delay in early hydration rate was observed in fly ash blended cement.

## 5 Hardened properties

**Fig. 23:**
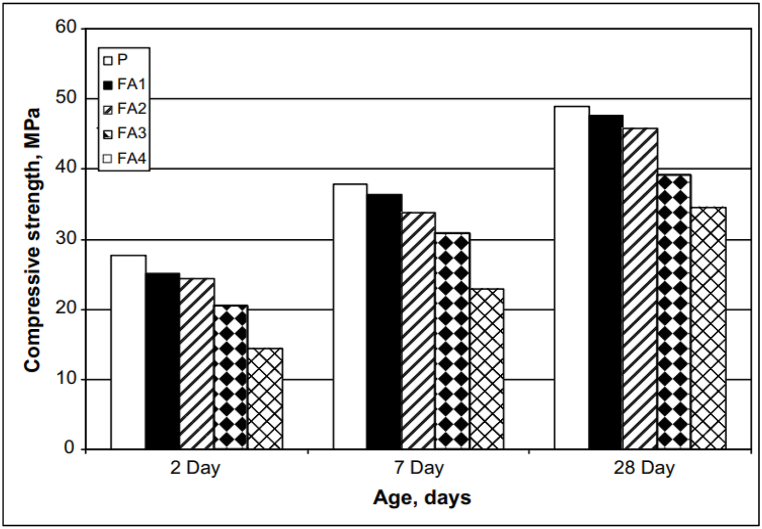
Compressive strength of fly ash blended mortar [46]. P- Control Mix, FA1 - 5% FA + 95% OPC, FA2 - 10% FA + 90% OPC, FA3 - 20% FA + 80% OPC, FA4 - 40% FA + 60% OPC.

Portland cement mortar hardens relatively quickly and also attains a high strength but is infamous for its poor early age properties. In the case of FA-blended cement (see Fig. 23), as the replacement of FA increased, the compressive strength decreased. Work by Zhang et al. [60] studied the effect of the architecture of PCE-based superplasticizer on the early strength of cement mortar. It revealed that at a water binder ratio of 0.3, polymers with longer side chains are advantageous for early strength development. However, Ozen et al. [71], in their study concluded that compressive strength of cement paste was not affected by side chain length of admixture. Saric-Coric et al. [86] concluded that the compressive strength of mortar is related to the degree of hydration and pore volume. The authors compared the compressive strength of mortar with PMS- and PNS-based superplasticizer. The mortar with PMS-based superplasticizer achieved the highest 7-day and 28-day strengths, nearly 20% more than its PNS-based superplasticizer counterpart. As it is well known that FA blended cementitious system has a downside of low early strength development and unpredictable long-term performance of concrete [47]. Replacement levels up to 15% have shown similar strength to the OPC but decreased with further addition (Fig. 23). This problem is usually associated with low-calcium fly ash (FAL, CaO less than 10%). The setback can be greatly reduced by using high-calcium fly ash (FAH, CaO greater than 20%), which is a residue left after the combustion of sub-bituminous coal in thermal power plants. The work of Papadakis [56], who first pulverised FAH, which parted the cenosphere into smaller particles in the plerosheres, eventually decreased the glass phase from 65% to 50%. Further, the author replaced FAH with aggregate (10%, 20%, and 30% by weight) and cement (20% by weight). The author finally concludes that for all replacement levels of FAH with aggregate, the compressive strength of mortar was significantly higher than control specimens. Also, for specimens with 20% replacement of FAH as aggregate, it was observed that strength gain was about 31% in the first 3 days, which indicated good pozzolanic-cementitious activity taking place during hydration. Overall, the use of a superplasticizer in optimum dosage helps to improve the compressive strength of cementitious matrix and reduces the water-binder ratio significantly. For FA blended cement, a replacement level of 10–15% gives improved strength performance. Using FA blended cement with any type of superplasticizer has less to do with the properties of the superplasticizer and more to do with the properties of FA, such as CaO and cenosphere content.

**Fig. 24:**
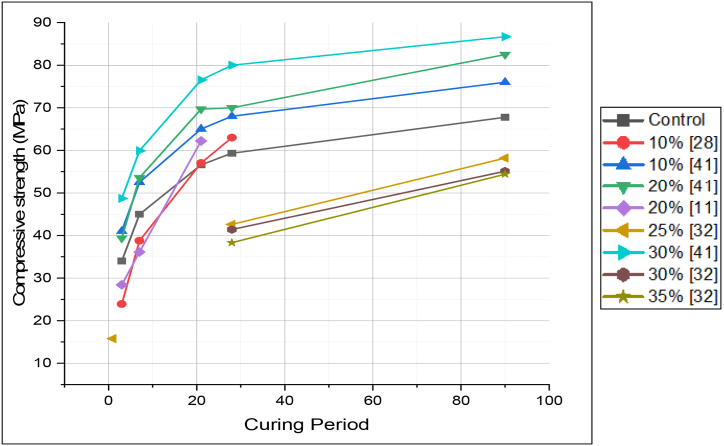
Compressive strength of fly ash blended cement (with PCE-based superplasticizer) at different replacement levels.

The work of Danish and Mosaberpanah [11] clearly highlighted the shortcoming of FA blended cement. The authors replaced FA and cenosphere at different percentages and found that the compressive strength of mortar at 3, 7 and 28 days was less than control mix. The reason for such behaviour was the hollow structure of cenosphere, which cracked under loading. Also, to a certain extent, the reaction between silica (from the cenosphere) and calcium hydroxide (from cement hydration) resulted in C–S–H gel formation. But as calcium hydroxide dissipated, the compressive strength stagnated. Erdoğdu et al. [45] used PMS-based superplasticizer to retain slump for duration of 90 min, and found that FA blended concrete showed slight increased compressive strength at 28 days as compared to plain concrete. The authors explained that, due to the addition of a superplasticizer, the slump could be retained for a longer duration, which decreased the air content in the concrete mix. Fig. 24 demonstrates the compressive strength of FA blended cement at different replacement levels.

**Fig. 25:**
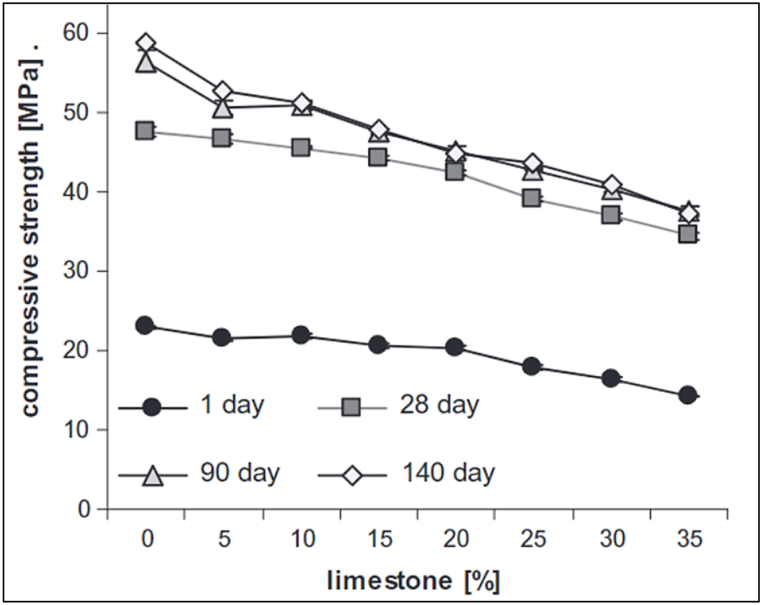
Compressive strength for curing of 1, 28,90 and 140 days for Limestone blended cement [47].

**Fig. 26:**
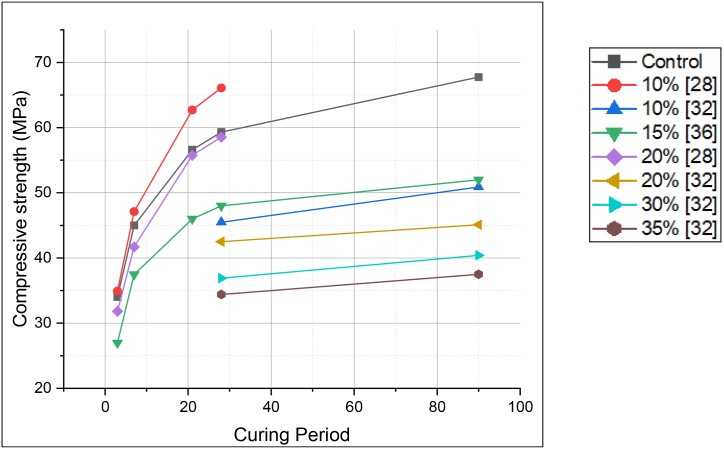
Compressive strength of Limestone blended cement (with PCE-based superplasticizer) at different replacement levels.

It was generally stated that LS blended cement showed higher early-age compressive strength. However, Weerdt et al. [47] stated that with an increase in LS replacement, the compressive strength decreased. For the first 90 days, the compressive strength seems to increase, as illustrated in Fig. 25. Although when comparing LS blended cement with FA blended cement for the same replacement level and curing period, the former showed slightly higher strength development. Uysal et al. [48] suggested that this could be due to limestone mostly acting as an inert filler. Fig. 26 demonstrates the compressive strength of LS blended cement at different replacement levels.

Vance et al. [43] studied the effect of fineness of LS in blended cement, and found that LS powder with nominal median particle size (0.7 and 3 μm) showed, good early age strength. Strength of blended cement containing 10% replacement of OPC with LS powder of 0.7 μm nominal median particle size gives highest strength at 14 days. Lin et al. [84] in their study found that LS-MK blend (5% LS and 7.5% MK) provided compressive strength of nearly 54 MPa (at 28 days) due to synergic effect of pozzolanic reactivity of both the SCMs.

**Fig. 27:**
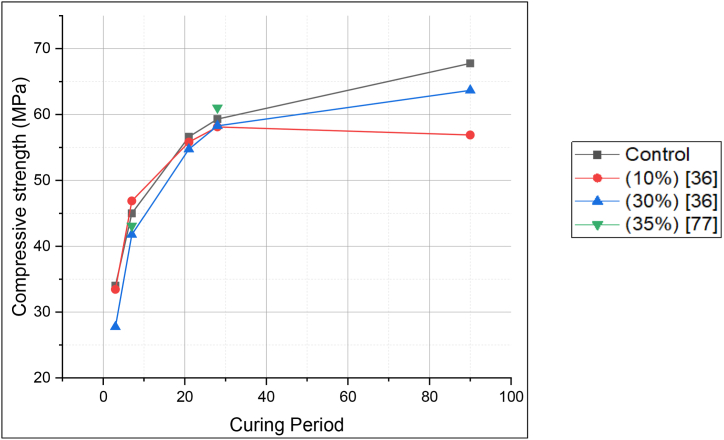
Compressive strength of Metakaolin blended cement (with PCE-based superplasticizer) at different replacement levels.

The addition of MK to cement improves the compressive strength of the cementitious system. This increase in strength was attributed to the behaviour of metakaolin as a mineral admixture, which makes the microstructure denser [16,92]. Due to the addition of metakaolin; total porosity, transitional pore and capillary pores were significantly reduced. Fig. 27 demonstrates the compressive strength of MK blended cement at different replacement levels. Use of MK blended cement has shown promising results both with and without superplasticizers, but not so with LS blended cement, as LS generally works as filler material in the cementitious matrix.

Early age compressive strength of GGBFS blended cement was not as good as Portland cement due to lower pozzolanic reactivity but at 28 days the compressive was more than control. As explained by Li and Ding [55], this was because the reactivity of GGBFS depends on the availability of portlandite (CH) and alkali from porltand cement to activate it. Uysal et al. [48] suggested that the presence of lime in pozzolanic material caused secondary hydration, which helped improve strength at later stages. A study conducted by Kanchanason and Plank [91] showed that when PCE-based superplasticizer (2% by wt. of cement) was added to GGBFS cement, the strength development was accelerated significantly. The GGBFS cement with PCE-based superplasticizer demonstrated a 25–95% increase in the compressive strength of mortar overall.

## 6 Microstructural analysis

### 6.1 Thermogravimetric analysis (TGA)

**Fig. 28:**
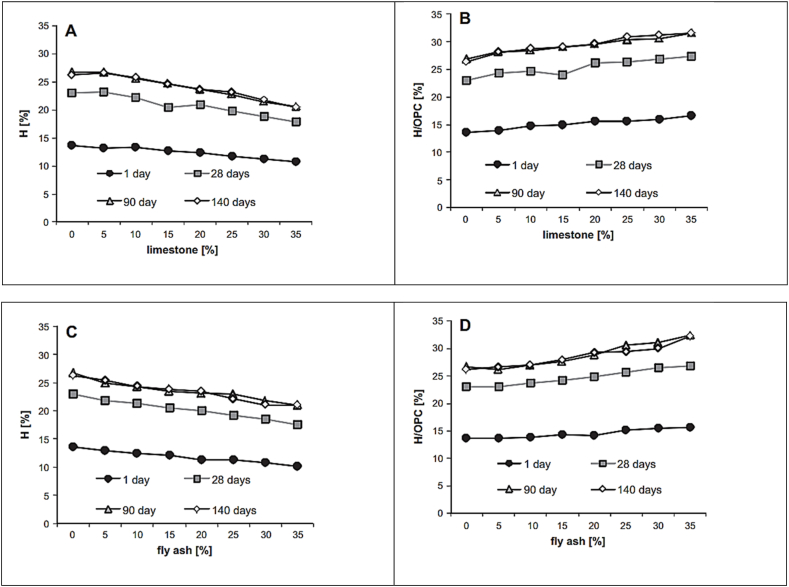
Amount of bound water (H) relative to dry content and OPC at curing period of 1,28,90 and 140 days for: (A) and (B) limestone blended cement and (C) and (D) for fly ash blended cement [47].

Thermogravimetric analysis (TGA) is a technique used to determine the thermal stability of materials. The technique monitors the weight change that occurs when the sample is heated at a constant rate. Generally speaking, bound water with respect to dry content decreased for blended cement, but relative to OPC, bound water increased. This phenomenon is common across FA and LS blended cements, as shown in Fig. 28. Weerdt et al. [47] indicated in their study that CH content (per dry content) decreases as replacement levels of FA increase. This effect is more prominent for replacement levels above 20%.

**Fig. 29:**
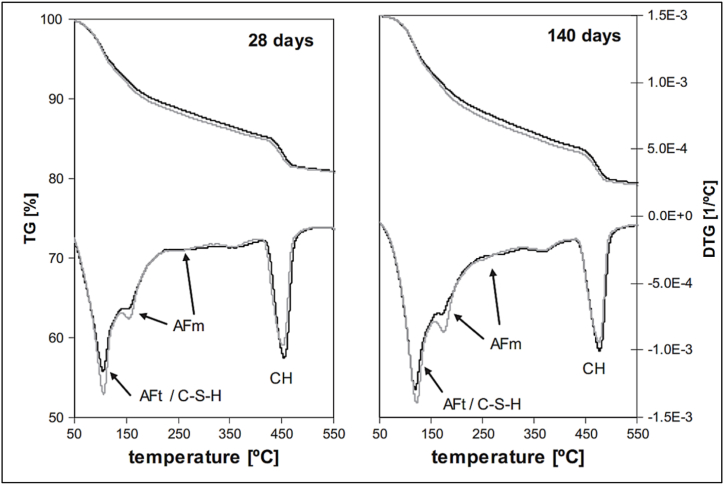
Thermogravimetric (TG) and differential thermogravimetric (DTG) of Portland cement (dark grey line) and 5% Limestone + 95% OPC (light grey line) after curing of 28 days and 140 days [47].

**Fig. 30:**
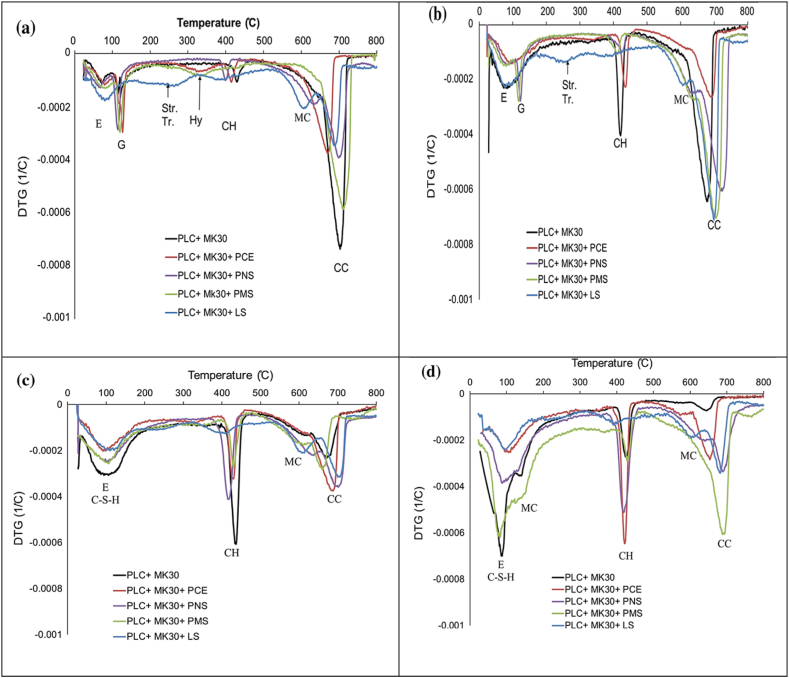
TGA of limestone-metakaolin blended cement with different superplasticizer at hydration of (a) 2 h, (b) 6 h, (c) 12 h and (d) 48 h. E: ettringite, G: gypsum, Str.: Stratlingite, Tr.: Tetra calcium aluminate hydrates, MC: monocarboaluminate [50].

The hydration reaction of MK is much faster as it has a slack microstructure. TGA of MK blended cement alone showed that MK reacted with calcium carbonate to form hemi-carboaluminate and also reacted with portlandite to form C–S–H gel. Results from Weerdt et al. [47] showed a clear peak at 100^o^C, suggesting the decomposition of the AF_t_ phase (ettringite). It appeared that the LS stabilised ettringite. A small nudge at 180 °C suggested decomposition of AF_m_ phases (Fig. 29). 2 h after the preparation of the paste with PCE-based and PNS-based superplasticizer, calcium hydroxide (CH) was found. Whereas gypsum was not present in the paste with the LS-based superplasticizer at 2 h due to the formation of calcium sulfoaluminate like ettringite. LS-based superplasticizer was also responsible for retarding the conversion of hexagonal C_2_AH_8_ to the hydrogarnet phase (C_3_ASH_6_). It is evident from Fig. 30 that paste containing PCE-based or PMS-based superplasticizers showed enhanced metakaolin reactivity, leading to the formation of hydrogarnet phases. In general, superplasticizers retarded the formation of CH in pastes containing MK (except for LS-based superplasticizers), which further delayed the hydration of C–S–H. This was because the superplasticizer adsorbed on cement particles and formed calcium complexes at the surface, which retarded the pozzolanic reactivity of MK at an early stage [50].

### 6.2 Scanning electron Microscope (SEM)

**Fig. 31:**
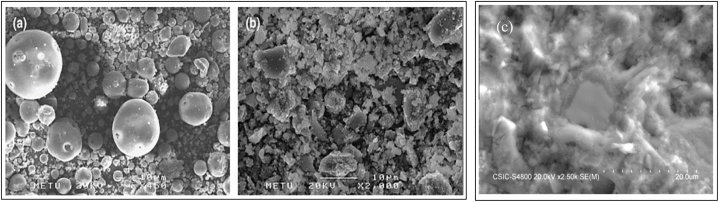
SEM images (a) smooth texture and round FA particles [94] (b) Smaller size LP particles [94] (c) 50% limestone added cement paste [49].

**Fig. 32:**
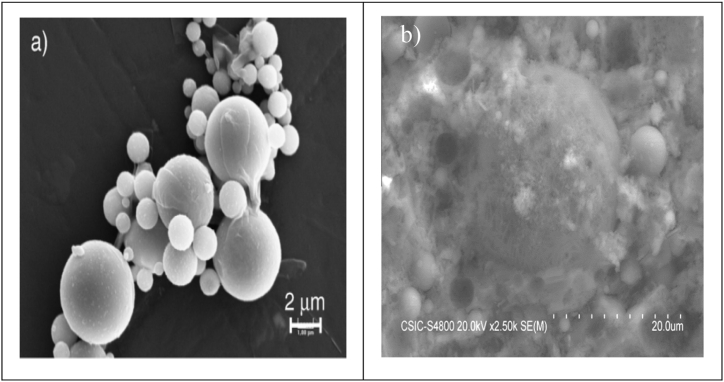
(a) SEM-micrograph of fly ash [44], (b) SEM micrograph of 50% limestone blended cement paste cured for 28 days [49].

The SEM technique is used to observe the surface of any solid material. A beam of electrons is emitted, which interacts with the atoms in the sample and produces a surface topography. The SEM observation of two cement pastes with and without superplasticizer at seven days was observed [93]. Ca, Al, and S were clearly detected in both cement pastes in the energy dispersive X‐ray analysis (EDAX) diagram, showing that both cement pastes were made up of analogous primary ions. The extra Na ions were detected because of the superplasticizer. Apart from this, a great delay in setting time was observed because of the extensive reactivity of the C_3_A, the superplasticizer present and the limited amount of C_3_A. At the start of the hydration phase, C_3_A released a remarkable amount of Al(OH)^4-^, which developed ettringite crystals. These ettringite crystals consumed all available superplasticizer particles [93]. The SEM analysis of different superplasticizers and mineral additives, namely LP powder and FA, incorporated in cement paste was examined. It was observed that FA particles were coarser than LP particles as shown in Fig. 31(b). FA particles had a smooth surface texture and relatively spherical geometry, shown in Fig. 31 (a), reducing their surface area to adsorb more free water [94]. Burgos-Montes et al. [49] studied the SEM analysis of 50% LS added cement paste at 28 days (Fig. 31 (c)). It showed that LP particles developed an essential phase of cement paste and also produced a very dense and compact microstructure, as shown in Fig. 32. This compact development did not significantly contribute to the strength development and formation of more cohesive products, but it helped in the distribution and dispersion of particles. In case of high proportion of FA (50%), the amount of water was very less as compared to the ash present. This caused a restriction in the pozzolanic reaction in many of the particles [95]. SEM was also used to observe the morphology of phases in different blended cement that showed pronounced formation of ettringite at early stage of hydration. By adding a superplasticizer, it was observed that there was a clear modification in the formation of ettringite for all blended cement pastes.

### 6.3 X-ray diffraction (XRD)

**Fig. 33:**
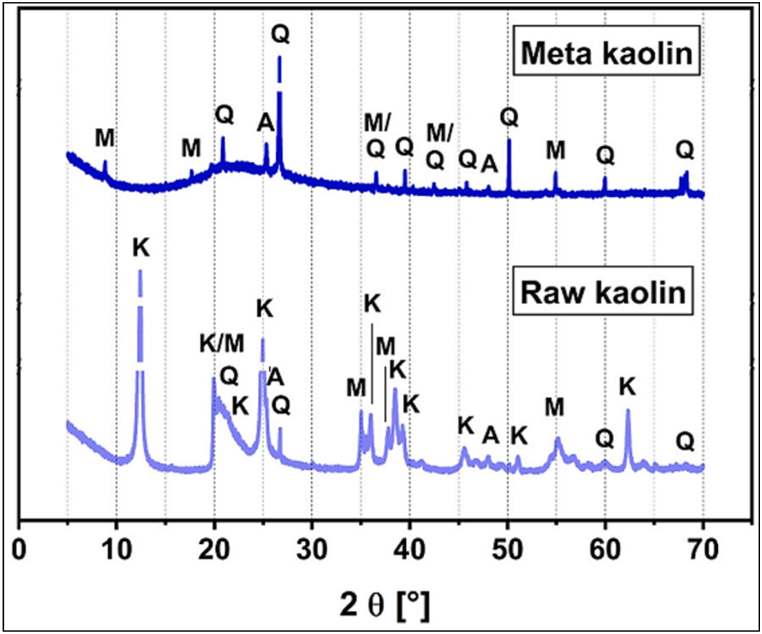
XRD of raw kaolin and metakaolin clay [75].

XRD is a popular technique used to primarily characterize material properties such as crystallite size and crystal structure. XRD of Portland cement on 1, 7, 28, and 90 days showed the formation of mono-sulfoaluminate, ettringite, and a rising quantity of portlandite [51]. Hydration products such as ettringite and strätlingite with mono-sulfoaluminate were present in MK blended cement after 7, 28, and 90 days [51]. Fig. 33 depicts XRD patterns for kaolinite clay and metakaolin clay. However, quantity of portlandite was less compared to Portland cement. Whereas, when blended with LS, at 7 days, hemi-carboaluminates were observed, and at 28 days, mono-carboaluminates were observed.

**Fig. 34:**
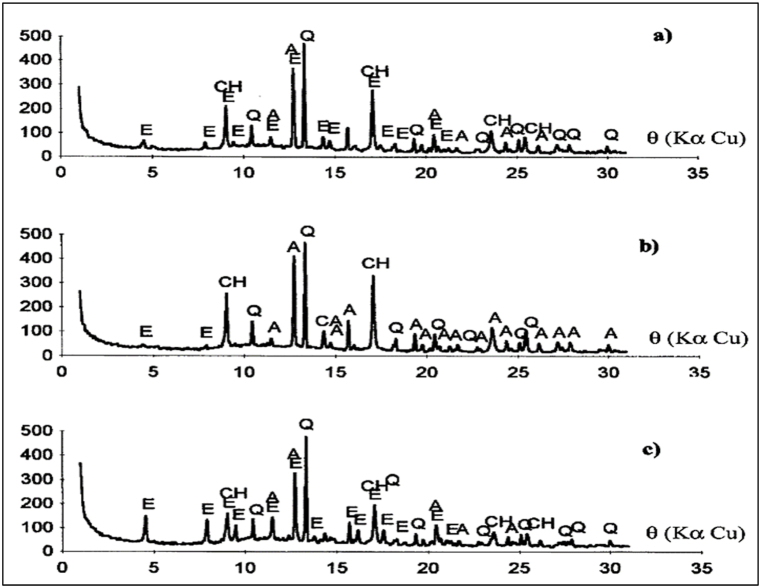
XRD pattern of superplasticizer added control cement paste containing (a) after 2 h of mixing (b) after 24 h of mixing (c) after 7 days of mixing. E = Ettringite; CH = Lime; A = Anhydrite; Q = Quartz [93].

As per the XRD pattern, the superplasticizer incorporated OPC paste clearly showed ettringite peaks at 2 h (Fig. 34 (a)), which were not observed at 24 h after mixing (Fig. 34 (b)). Between the first day and the fifth day, the ettringite peak was almost entirely missing. After the fifth day, it appeared again, and its peak intensity was gradually increased till the seventh day (Fig. 34 (c)). The crystallization noticed in OPC paste represented the end of setting time. It showed that a strong reaction takes place between the superplasticizer molecules and the initial ettringite. Apart from this anhydrite, quartz and lime phases were also detected in cement paste at all hydration periods [93].

**Fig. 35:**
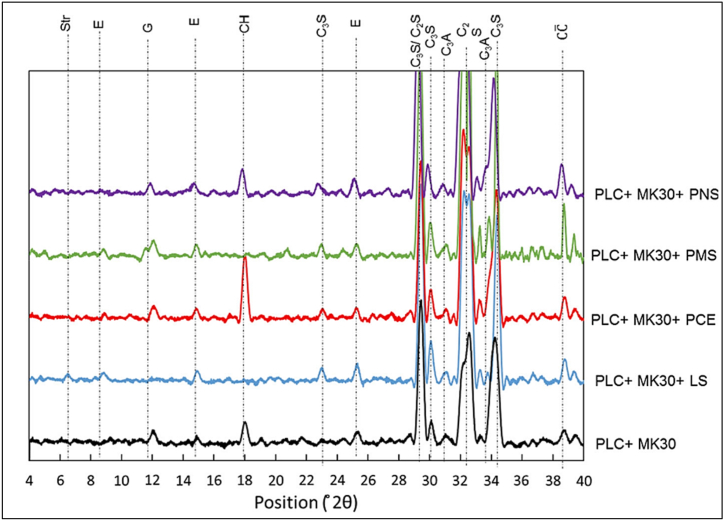
XRD of PLC + metakaolin (30%) with different type of superplasticizer at 6 h of hydration [50].

Li and Ding [55], in their study stated that portlandite (CH) peak was highest for control followed by MK blended cement with PNS-based superplasticizer at 28 days hydration period, as evident from Fig. 35. This was due to the reaction of MK with CH, which translates into C–S–H gel. MK, being a Class N pozzolan, reacts with lime and showcases cementitious properties. In a blended cement with the addition of PMS-based superplasticizer, the portlandite formation was lower at 6 h (compared to the control), therefore resulting in a lower C–S–H formation. But at 48 h, the paste exhibited a high ettringite content, suggesting hydration of calcium aluminate phases. For paste with PCE-based superplasticizer, at 48 h, portlandite content was increased but ettringite peak was lower than control [50]. Antoni et al. [51] inferred that the LS-MK blend enhanced the formation of ettringite, and a significant increase in hemi-carboaluminates formation was also observed (Fig. 35).

**Fig. 36:**
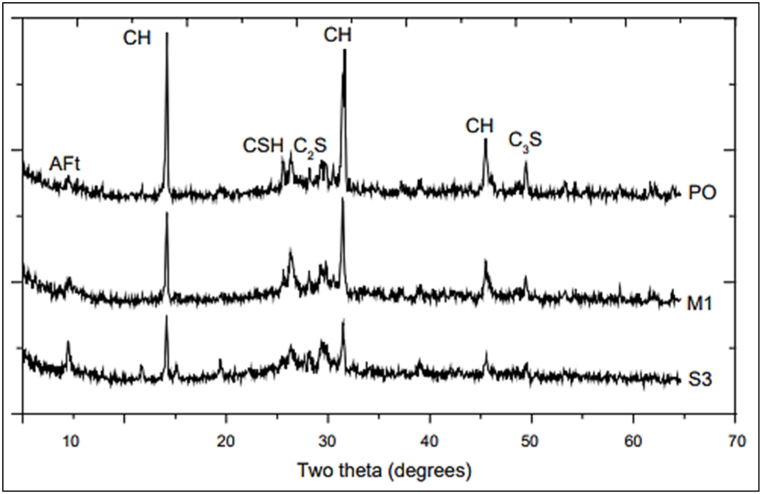
XRD of cement paste hydrated for 28 days. PO represents Portland cement and M1 represent 10% metakaolin blended cement (PO=OPC mortar; M1 = MK blended cement mortar; S1 = GGBFS blended cement mortar) [55].

XRD pattern of MK (10%) and GGBFS (20–30%) with two superplasticizers (naphthalene formaldehyde condensate and sulphonated) incorporated cement paste after 28 days of hydration was investigated. The product phase CH and Ca(OH)_2_ peaks were highest in control cement paste among other cement pastes, as shown in Fig. 36. Cement pastes with 10% MK and 30% GGBFS showed the lowest CH peak. This was caused by the reaction between the CH phase and the MK and GGBFS contents. CH did not directly contribute to strength development. After converting CH into C–S–H gels by pozzolanic reaction with active minerals, cement paste gained strength. Because C–S–H has a greater specific surface, which helped in strong compound force in cement paste. On the other hand, AF_t_ phase was also developed in blended cement [54].

**Fig. 37:**
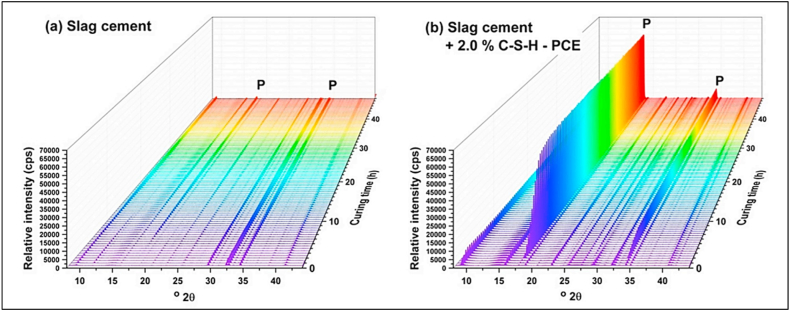
XRD patterns of (a) GGBFS cement and (b) GGBFS cement with PCE-based Superplasticizer (2% by wt. of cement). P: Portlandite at 18° and 34.1° 2θ [91].

Kanchanason and Plank [91], in their study concluded that GGBFS cement, in first 48 h of hydration showed formation of portlandite as by-product of silicate reaction. With PCE-based superplasticizer, it was observed that after 2 h of hydration, a profound reflection of portlandite occurred (Fig. 37), but soon the intensity was decreased, meaning the commencement of the pozzolanic reaction.

## 7 Durability

### 7.1 Water absorption

Water absorption is an important property as it affects various durability properties of cementitious materials. In the study conducted by Danish and Mosaberpanah [11], blended cement mortar with 20% FA showed more water absorption compared to the control mix due to the high surface area and fineness of FA particles. When the FA was blended with 5–10% cenosphere, the water absorption decreased by 12–16%, compared to 20% FA blended cement. Although it showed higher water absorption with respect to the control mix due to the porous nature of cenosphere particles, which resulted in the formation of more capillary pores.

**Fig. 38:**
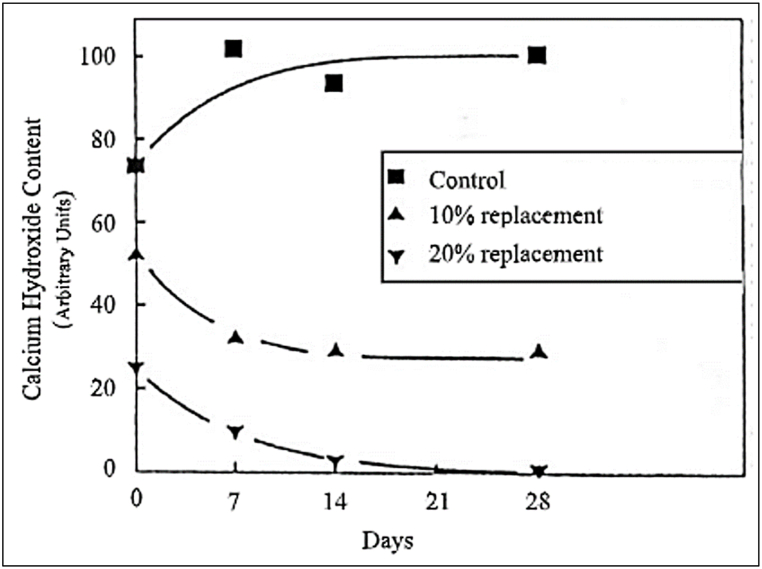
Variation of CH content in concrete with metakaolin replacement [83].

MK blended cement helps in converting portlandite (CH) to C–S–H, as CH is often linked to poor durability. Alumina and silica present in MK reacts with CH. With an increase in MK replacement levels, the CH content of hydrated cement drastically decreased, as evident from Fig. 38. In a water impermeability test conducted by Uysal et al. [48], the results showed that concrete with 20% GGBFS replacement showed lower imperbeability (in the range of 4.5–12.5 mm).

### 7.2 Chloride ion penetration

Concrete containing FA and GGBFS showed less chloride penetration due to the development of an intermittent pore structure and a dense microstructure. FA and GGBFS are alumina-rich and lead to a higher amount of C_3_A, which consequently leads to increased formation of calcium silicate hydrate (C–S–H). This C–S–H improved the chloride binding ability of concrete, thus making it better resistant to chloride ion penetration [48].

## 8 Recent research activity at CSIR-CBRI

Recently, the researchers at CSIR-Central Building Research Institute conducted the research and development on cementitious composites employing a variety of mineral admixtures/supplementary cementitious materials [[96], [97], [98], [99], [100], [101], [102], [103], [104], [105], [106]]. Anurag et al. [41], in their study on SCMs such as fly ash, silica fume, low-grade calcined limestone (CLS), low-grade uncalcined limestone (UCLS), and superplasticizer assessed various properties such as fineness, setting time, consistency, compressive strength, and flexural strength. The aim of the study was to determine the optimum dosage of uncalcined and calcined limestone that can enhance the physico-mechanical properties of cement mortar, and to study the effect of uncalcined and calcined limestone with other SCMs as well as superplasticizer. In the study, the authors concluded that SCMs have high pozzolanic activity. SCMs such as SF and MK exhibit better strength and a denser microstructure due to the high specific surface area of these SCMs when compared to others. Authors also concluded that the usage of low-grade limestone can reduce the environmental pollution and can be used in cement industry effectively.

Kumar et al. [107] studied the synergetic effect of hydroxypropyl methylcellulose (HPMC) on fly ash-incorporated OPC cement paste. The authors investigated properties such as consistency, setting time, hydration kinetics, compressive strength, and thermal-cum-microstructural properties. The authors found that HPMC enhances the workability of blended cement due to its viscosity-modifying properties. However, it was evident that FA and HPMC interaction hinders the hydration product formation, thus increasing the initial and final setting time.

**Fig. 39:**
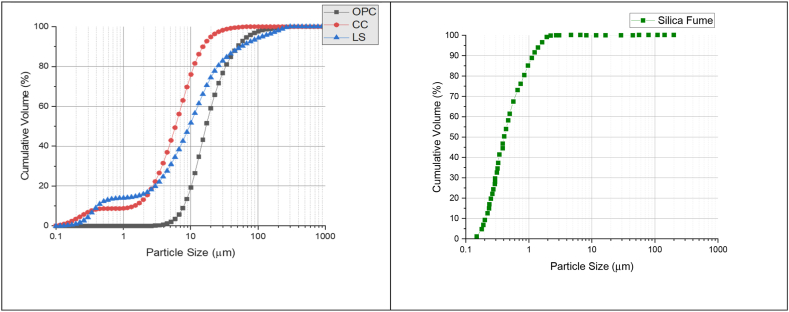
Particle size distribution for all four raw materials (D_50_ values for CC = 6.04 μm, LS = 11.17 μm, OPC = 20.48 μm and SF = 0.3 μm).

Currently, research work on the optimization of LC^3^SF along with PCE-HPMC has been done at CSIR-CBRI, Roorkee. The intention of the study was to develop high strength LC^3^SF with a target strength comparable to OPC (53 grade). Box-Behnken Design (BBD) was used for multi-objective optimization. Using Design Expert v.11 software, a total of 27 experiments with 3 replications of the central point were designed to allow pure error estimation. OPC-43 grade was acquired from M/s. Wonder Cement Limited, Chittorgarh, Rajasthan. Calcined Clay was acquired from M/s. Gujarat Earth Minerals Pvt. Ltd., Ahmedabad, Gujarat. Limestone in the form of Low-grade limestone slurry (Kota Stone) from M/s, Subhgiri Enterprise, Kota Rajasthan was grinded in ball mill to optimize the particle size distribution (PSD). SF was acquired from EVONIK, Aerosil® 200. It was a hydrophilic silica. Silica fume is an amorphous mineral material composed of extremely small, chemically active particles of SiO_2_ that behave as supplementary cementitious materials that improve the packing density of the final product. Two chemical admixtures were used for the study, namely, Poly-carboxylate ether-based superplasticizer (PCE), which was supplied by ShaliPlast® PCE 100, and Hydroxy-propyl methylcellulose (HPMC), which was supplied by M/s Loba Chemie Pvt. Ltd., India. The particle size distribution for all four raw materials is shown in Fig. 39.

**Fig. 40:**
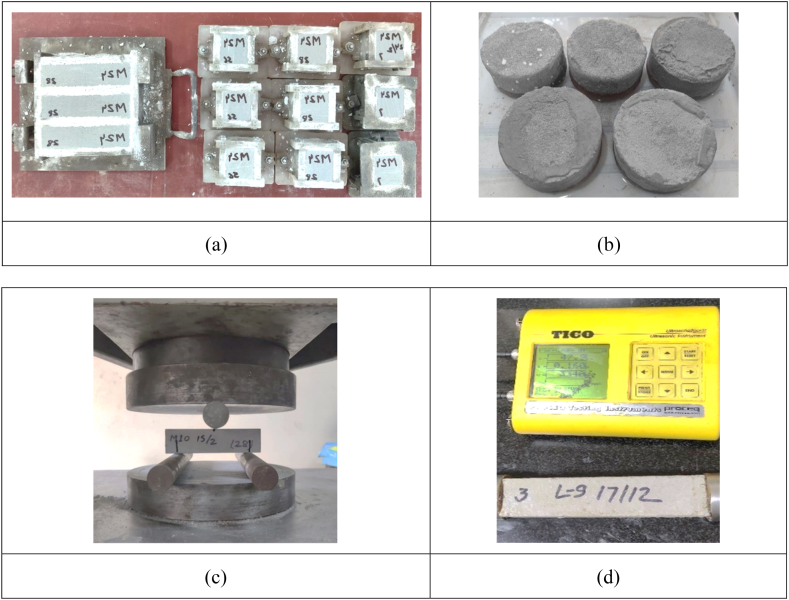
(a) Prims (160 mm × 40 mm × 40 mm) for MOR and cubes (50 mm × 50 mm × 50 mm) for CS. (b) Specimen (Dia. 100 mm and ht. 50 mm) for water sorptivity. (c) Three-point loading test was performed on a 20 KN Shimadzu universal testing machine and (d) UPV being performed on TICO Ultrasonic pulse velocity.

For the production of LC^3^SF , LC^3^ was replaced by 3% and 6% silica fume (by wt.) and to ensure a homogenised mix, the blend was interblended in the ball mill again for 15 min. Three-level-four (3^4^) factorial BBD (Design Expert® version 11) was used to study the impact of four input parameters (A: SF%, B: SP%, C: HPMC%, and D: Flow Value) on eight dependent outputs (Water binder ratio, Compressive strength (7 and 28 Days), Modulus of rupture (28 days), Porosity, Water absorption, Water sorptivity and Ultra-sonic pulse velocity). Replacement levels for SF with LC^3^ were 0%, 3%, and 6%. While PCE and HPMC were added at varying levels of 0%, 0.5%, and 1% by wt. of binder. The flow value variation was 120 mm, 140 mm, and 160 mm. A total of 27 mortar mixes were cast along with the control mortar (OPC 100%). For CS (7 and 28 days), 3 cubes each of size 50 mm were cast as per IS 4031, Part 6: 1988 [108] and tested on a 1000 kN Shimadzu universal testing machine. For MOR, 3 specimens of size 160 mm × 40 mm x 40 mm per mix were cast as per ASTM C348–21 [109] and tested on a 20 kN Shimadzu universal testing machine. Porosity and water absorption were performed as per ASTM C642–21 [110]. Water sorptivity was performed on a specimen with a diameter of 100 mm and a height of 50 mm. UPV was performed on specimen of length 160 mm according to IS 13311 (1) – 1992 [111]. The instrument used for this test was TICO Ultrasonic pulse velocity with specifications, measuring range: 15–6550 μs; frequency: 70 kHz and frequency depth up to 3 m (Fig. 40).

Response surface methodology (RSM) involves modelling and analysis of problems using mathematical and statistical techniques. For finding the statistical relationship between input parameters and output responses, ANOVA analysis was done. This analysis determines the sum of squares, mean, lack of fit, F-value, and p-value. The results of the ANOVA predict that the significant model was quadratic for all responses. Statistical analysis (ANOVA) showed that R^2^ varies from 0.94 to 0.98, which is desirable for a perfect model.

After analysis of the model by ANOVA, a multi-objective-based optimization technique was applied to find the optimised mix with the optimum admixture dosage and desirable compressive strength. Based on RSM and the desirability approach, optimization was done by setting the range of output responses according to target-desirability and maintaining the desired range of input parameters, as shown in Table 5.

**Table 5:** Optimization of input parameters and responses

Name	Goal	Lower Limit	Upper Limit	Lower Weight	Upper Weight	Importance
SF	is in range	0	6	1	1	3
SP	is in range	0	1	1	1	3
HPMC	is in range	0	1	1	1	3
FV	is in range	120	160	1	1	3
w/b ratio	minimize	0.4	0.55	1	1	3
CS (7 days)	maximize	33	36	1	1	3
CS (28 days)	maximize	43	60	1	1	3
MOR	is target = 10	7	12	1	1	3
Porosity	minimize	2	4	1	1	3
Water absorption	minimize	1	10	1	1	3
UPV	minimize	4300	4500	1	1	3
Sorptivity	minimize	0.02	0.04	1	1	3

**Fig. 41:**
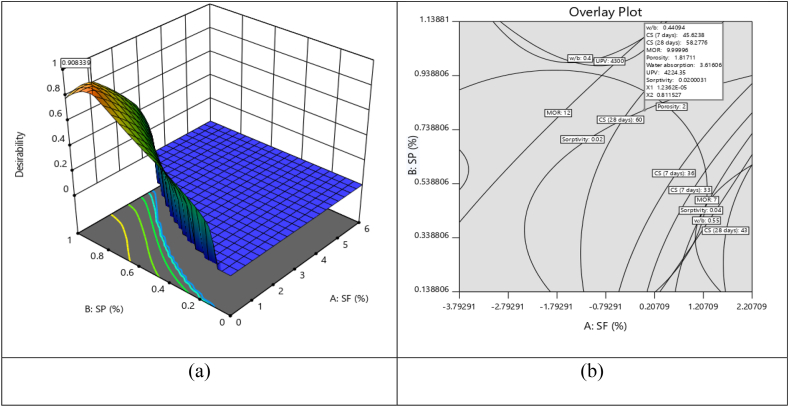
(a) 3D response of overall desirability and (b) Overlay plot after optimization of parameters.

**Fig. 42:**
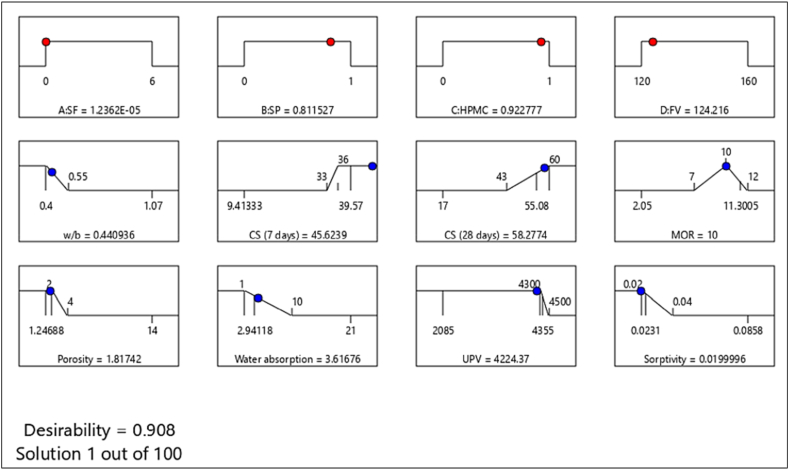
Ramp of desirability for optimised mix.

After optimization, 100 solutions were obtained with varying desirability. Desirability of 1 or closer to 1 is preferable for obtaining the best results with effective use of input parameters, as shown in Fig. 41, Fig. 42. In our model, suitable desirability was obtained as 0.908. The results obtained from the model were water-binder ratio: 0.44, compressive strength (7 days): 45.64 MPa, compressive strength (28 days): 58.27 MPa, MOR: 10 MPa, Porosity: 1.81%, Water absorption: 3.61%, UPV: 4224 m/s, and sorptivity: 0.02 mm/min^1/2^. To validate the results obtained, the selected optimised mix was cast, and experimental results were compared with model response values. To validate the optimised model, residual standard error (RSE) is calculated for all output responses. In this study, all the RSE values were less than 10%.

The aim of the research was to achieve strength comparable to OPC-53 grade with mineral and chemical admixture-incorporated Limestone calcined clay cement (OPC-43 Based). Also the secondary aim was to minimize water-binder ratio (lower limit: 0.40 and upper limit: 0.55), maximize compressive strength (7 days) (lower limit: 33 MPa and upper limit: 36 MPa), maximize compressive strength (28 days) (lower limit: 43 MPa and upper limit: 60 MPa), in range 28 days-MOR (lower limit: 7 MPa and upper limit: 12 MPa), minimize porosity (lower limit: 2% and upper limit: 4%), minimize water absorption (lower limit: 1% and upper limit: 10%), minimize UPV (lower limit: 4300 m/s and upper limit: 4500 m/s) and minimize sorptivity (lower limit: 0.02 mm/min1/2 and upper limit: 0.04 mm/min^1/2^). Based on statistical modelling, all the models were found statistically significant according to p-value, lack of fit, and R^2^. The R^2^ value for all 8 responses was in the range of 0.94–0.98, which is necessary for a perfect model. Using the perturbation plot, it was observed that as SF% increases, the water-binder ratio, porosity, water absorption, and water sorptivity also increase, but compressive strength (7 and 28 days), Modulus of rupture (28 days), and ultrasonic pulse velocity decrease. It was observed that there was little to no effect of HPMC on compressive strength. But when flow value was decreased from 160 mm to 120 mm the compressive strength (28 days) increased by nearly 80% [112].

## 9 Research gaps

The following research gaps were observed:

1. The impact of admixtures, and mainly superplasticizers, on this flocculation process in LC^3^ cements still remains unknown.
2. In the case of calcined clay cements, fluidity retention is one of the main limitations that needs to be overcome, and the mechanisms involved in their rapid flow loss still remain unknown.
3. The amount of unabsorbed superplasticizer and its effect on hardened properties and microstructural development are little known.
4. Influence of clay impurities in aggregate and its effect on efficiency of superplasticizer.
5. Experimental characterization still remains a challenge as multiparameter studies are necessary, including revealing the role of the polymer molecular structure, the nature and content of the SCMs, the specific surface area of the solids and their surface charge, as well as the chemical composition of the pore solution.
6. More studies are required to understand the main mechanism responsible for the fast flow loss of superplasticizer in calcined clay cements.

## 10 Conclusions

In the quest to achieve higher substitution levels, reduce environmental emissions and ecological impact, blended systems are extensively studied for their mechanical and durability properties. However, our knowledge about how these SCMs cements react with superplasticizers and their impact on fresh, hardened, and microstructural properties is insufficient. In this paper, the interaction of blended cement with superplasticizer was studied. To sum up, the following conclusions can be drawn:

1. It is expected that superplasticizer will lower the water demand while maintaining workability of blended cement. Especially PCE-based superplasticizers are more effective as their absorption by blended cement particles is often higher. High performance concrete like Self compacting concrete (SCC), high-fluidity concrete and high-performance concrete can be achieved by using advance superplasticizers.
2. A higher dose of superplasticizer is required for blended cement, especially LS-MK blended cement. Although workability at saturation dosages of superplasticizer is similar for all binder systems.
3. Superplasticizer chemistry have significant consequences for the hydration kinetics and phase development of blended cement.
4. Replacement of LS up to 10% improves compressive strength, and up to 30% replacement, the rheology is the same as that of control paste.
5. The complex interaction of LS and superplasticizer improves packing density and mechanical properties. It also alleviates the long-term strength loss problem significantly.
6. LS blended cement with a superplasticizer was found to accelerate the hydration of C_3_A significantly while delaying the C_3_S reaction.
7. The addition of 10–15% FA is suitable as it increases the compressive strength by 15–30% of the cementitious matrix.
8. When Portland cement is replaced by FA, the final strength can exceed that of the control only if the content of active silica in the FA is higher than that in the cement.
9. Due to the low lime content in FA, the hydration rate was reduced, resulting in reduced drying shrinkage.
10. Using FA in concrete, the slump retention capacity initially reduces, but by incorporating a superplasticizer in the mix, the slump is restored to its initial condition and the compressive strength is also improved.
11. Due to the large specific surface area of MK, the superplasticizer absorption per unit surface area decreases significantly, and thus the workability of the cementitious mix tends to worsen.
12. Addition is 40% GGBFS increases the compressive strength but beyond that the strength decreases.
13. Among all the blended cement, FA and GGBFS blended concrete having superplasticizer showed lowest chloride ion permeability with less than 2000 coulombs of total charge passing.
14. The environmental impact of the cement industry needs to be assessed, as production of cement is a very complex process that requires enormous amounts of raw materials. Regardless of different functional groups, it is observed that cementitious matrix containing FA showed better environmental performance.

Resourceful use of SCMs in cement production provides an attractive option from a resource and cost conservation point of view. Moreover, with the global push for sustainability, the cement industry is under increasing pressure to reduce its dependency on natural resources. However, further research work considering the development of blended cement, chemical admixture compatibility, and mix design methods is needed for their commercial usage.

## Declarations

### Author contribution statement

**Ishan Bhandari:** Conceptualization, Data Curation, Validation, Visualization, Reproduction of results, Writing- original draft, Writing – review & editing. **Rajesh Kumar:** Funding acquisition, Conceptualization, Methodology, Reproduction of results, Application of statistical techniques, Creation of models, Writing – review & editing, Project administration, Supervision. **A Sofi:** Supervision. **Nikhil Nighot:** Writing – original draft, Writing – review & editing.

### Data availability statement

Data will be made available on request.

## Declaration of competing interest

The authors declare that they have no known competing financial interests or personal relationships that could have appeared to influence the work reported in this paper.
